# Yield Trends Are Insufficient to Double Global Crop Production by 2050

**DOI:** 10.1371/journal.pone.0066428

**Published:** 2013-06-19

**Authors:** Deepak K. Ray, Nathaniel D. Mueller, Paul C. West, Jonathan A. Foley

**Affiliations:** Institute on the Environment (IonE), University of Minnesota, Saint Paul, Minnesota, United States of America; New York State Museum, United States of America

## Abstract

Several studies have shown that global crop production needs to double by 2050 to meet the projected demands from rising population, diet shifts, and increasing biofuels consumption. Boosting crop yields to meet these rising demands, rather than clearing more land for agriculture has been highlighted as a preferred solution to meet this goal. However, we first need to understand how crop yields are changing globally, and whether we are on track to double production by 2050. Using ∼2.5 million agricultural statistics, collected for ∼13,500 political units across the world, we track four key global crops—maize, rice, wheat, and soybean—that currently produce nearly two-thirds of global agricultural calories. We find that yields in these top four crops are increasing at 1.6%, 1.0%, 0.9%, and 1.3% per year, non-compounding rates, respectively, which is less than the 2.4% per year rate required to double global production by 2050. At these rates global production in these crops would increase by ∼67%, ∼42%, ∼38%, and ∼55%, respectively, which is far below what is needed to meet projected demands in 2050. We present detailed maps to identify where rates must be increased to boost crop production and meet rising demands.

## Introduction

The world is experiencing rising demands for crop production, stemming from three key forces: increasing human population, meat and dairy consumption from growing affluence, and biofuel consumption [1]–[5]. By 2050, global agricultural production may need to be increased by 60%–110% to meet these increasing demands [3], [6]–[7] as well as to provide food security to the ∼870 million now chronically undernourished [8]. The only peer-reviewed estimate [3] suggests that crop demand may increase by 100%–110% between 2005 and 2050. Numerous authors have suggested that increasing crop yields, rather than clearing more land for food production, is the most sustainable path for food security [2], [4], [9]–[14]. Moreover, crop yield growth has been shown as an effective tool in reducing global poverty and undernourishment, as farmers themselves constitute the vast majority of the poor and the undernourished [15]–[17].

However, several recent studies indicate that yields may no longer be increasing in different regions of the globe [18]–[23]. Yields are no longer improving on 24–39% of our most important cropland areas [23]. Many of these areas are in top crop producing nations, having rising population, increasing affluence, or some combination of these factors [3], [5], [19], [21]–[23]. This may increase difficulty of meeting future crop production goals but key unknowns remain for developing and targeting strategies: how are crop yields changing across the world, where gains in crop yields are able to meet growing demands, and where crop yields are falling behind.

Here we employ ∼2.5 million statistics from a newly developed crop yield and area harvested database covering ∼13,500 political units globally from 1961 to 2008, focusing on trends in the recent two decades [4], [23]. We determine the rates of yield change in each political unit for the top four global crops: maize, rice, wheat, and soybean. These four crops together produce about two-thirds of current harvested global crop calories [3], [18]. Using these data, we estimate the best-fit linear, non-compounding rates of yield change between 1989 and 2008 for these crops in each of these political units. Yield change is commonly modeled as a linear function of time [24]–[28] and such models have been used to project future crop yields [28]–[30]. We provide local, country, and global-scale rates of recent crop yield changes to determine where the rates of yield increase could double production by 2050, and where they are insufficient. The impact of negative or even slow rates of yield change in these crops could be severe, especially for low-income countries with rapidly rising population. The underlying data, period analyzed, statistical approach, and comparisons of yield projections are described in the Methods section below, with additional details and analysis in Text S1.

## Results

**Figure 1:**
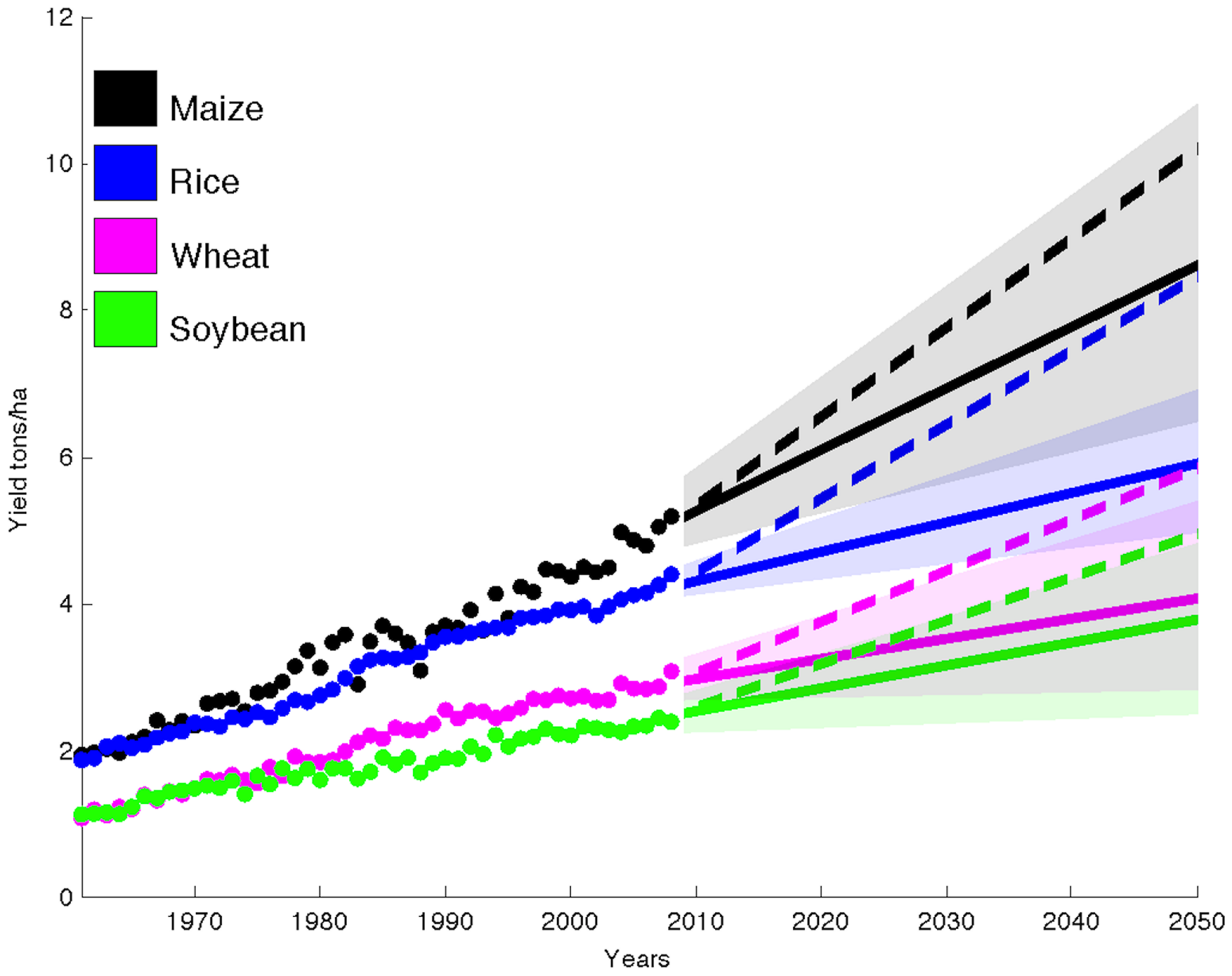
Global projections. Observed area-weighted global yield 1961–2008 shown using closed circles and projections to 2050 using solid lines for maize, rice, wheat, and soybean. Shading shows the 90% confidence region derived from 99 bootstrapped samples. The dashed line shows the trend of the ∼2.4% yield improvement required each year to double production in these crops by 2050 without bringing additional land under cultivation starting in the base year of 2008.

The global average rates of yield increase across ∼13,500 political units are 1.6%, 1.0%, 0.9%, and 1.3% per year for maize, rice, wheat, and soybean, respectively (Table 1, Figure 1). A ∼2.4% per year rate of yield gains (non-compounding) is needed to double crop production by 2050. Current rates are thus not achieving this goal. At current rates only ∼67%, ∼42%, ∼38%, and ∼55% increases in maize, rice, wheat and soybean production, respectively, is possible by 2050.

**Table 1:** Global summary for maize, rice, wheat, and soybean.

	MAIZE	RICE	WHEAT	SOYBEAN
Mean yield change per year (% per year)	1.6	1.0	0.9	1.3
Mean yield change per year (kg/ha/year/year)	84	40	27	31
Projected average yield in 2025 (tons/ha/year)	6.5	4.9	3.4	3.0
Projected average yield in 2050 (tons/ha/year)	8.6	5.9	4.1	3.8
Projected production in 2025 (million tons/year) at fixed crop harvested areas of 2008	1016	760	741	275
Projected production in 2050 (million tons/year) at fixed crop harvested areas of 2008	1343	915	891	347
Projected production shortfall in 2025, as compared to the rate that doubles production by 2050 (million tons/year)	100	160	157	43
Projected production shortfall in 2050, as compared to the rate that doubles production by 2050 (million tons/year)	247	394	388	107
Required extra land (million hectares) to produce the shortfall at 2025 projected yields	15	33	46	14
Required extra land (million hectares) to produce the shortfall at 2050 projected yields	29	67	95	28
Yield in the year 2008 (tons/ha/year)	5.2	4.4	3.1	2.4
90 percent confidence limit in yield change (%/year)	0.8–2.4	0.5–1.4	0.1–1.8	0.3–2.0
90 percent confidence limit in yield change (kg/ha/year/year)	41–124	21–58	4–52	6–50
90 percent confidence limit in production in 2025 (million tons/year) at fixed crop harvested areas of 2008	848–1203	687–846	599–898	214–328
90 percent confidence limit in production in 2050 (million tons/year) at fixed crop harvested areas of 2008	1009–1686	769–1072	618–1182	228–442

As an example consider yields and production in 2025 – the short term – and numbers by 2050 due to current rates of yield change. See Supplementary Data file for yield change rates per country.

We provide a range of future yield estimates by bootstrap sampling crop yield data at each of the political units studied for the period 1989 to 2008. The upper bound of the 90% confidence interval (Table 1, Figure 1) presents a slightly more optimistic scenario, global yields increase at rates of 2.4%, 1.4%, 1.8%, and 2.0% per year for maize, rice, wheat, and soybean, respectively. Yield trends following this upper bound projection could lead to ∼101%, ∼59%, ∼76%, and ∼84% increased production in these crops, respectively. The lower bound of our confidence interval provides us with a “worst-case scenario,” wherein the global average yield of maize, rice, wheat, and soybean would increase at 0.8%, 0.5%, 0.1%, and 0.3% per year, respectively (Table 1, Figure 1). At these rates global production could only increase by ∼34%, ∼21%, ∼4%, and ∼13% for maize, rice, wheat, and soybean, respectively, by 2050. Further, the yield trajectory diverges, especially for rice and wheat from the 2.4% per year rate (Figure 1). See Figure S1 for spatial maps of r^2^ at each political unit and statistical diagnostic tests (Text S1 and Figures S2, S3, S4, S5, S6, S7, S8, S9).

In the short term, due to population increases from ∼6.7 billion in 2008 to ∼8.0 billion in 2025 [5], the 1.6% and 1.3% per year global maize and soybean yield improvements may result in no significant change to the per capita global maize and soybean harvests. However, by 2050 there could be an increase. The much lower rates of rice and wheat yield increases, 1.0% and 0.9% per year, respectively, may result in no change to the per capita rice and wheat harvests to 2050. Thus, if we are to boost the production in these top four global crops that are now responsible for directly providing ∼43% of the global dietary energy and ∼40% of its daily protein supply [31] from yield increases alone, we have to immediately determine where and exactly by how much yields are changing. To further understand the yield trend patterns, we also track the rates of yield change within ∼13,500 political units and report the results at the local and country scales.

**Figure 2:**
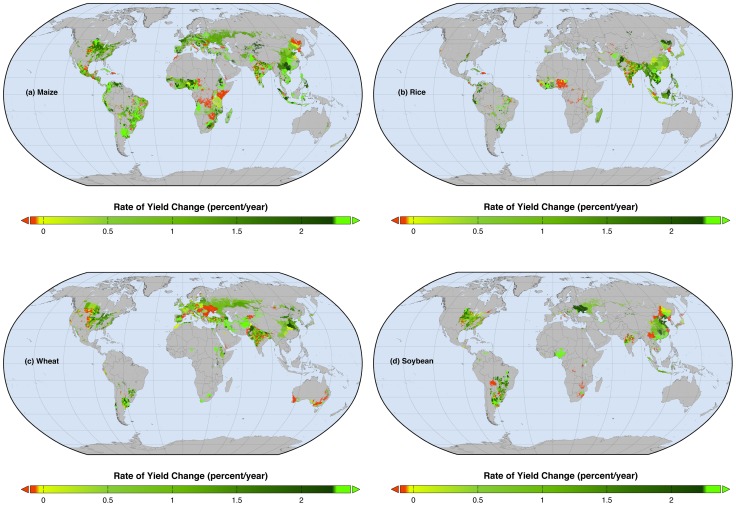
Maps of observed rates of percent yield changes per year. Global map of current percentage rates of changes in (a) maize, (b) rice, (c) wheat, and (d) soybean yields. Red areas show where yields are declining whereas the fluorescent green areas show where rates of yield increase – if sustained – would double production by 2050.

Global trends mask the significant variations in the rates of yield change among and within countries (Figure 2). We determine where the within-country yield change rates are ∼2.4% per year or above (i.e. doubling rates), where the rates are lower, and where yields are decreasing. We briefly describe these areas, emphasizing areas with doubling and decreasing rates as these areas define the places with the greatest opportunity to meeting growing demand or where to target investments. See Figure S10 for continuous rate (non-categorical) maps in kilograms/hectare/year/year. The influence of observed yields in 2008 on the percent rate of change is described in Figure S11 and related Text S1.

### North and Central America

Most of North Dakota and Mississippi, northeastern South Dakota, northwestern Minnesota, and some isolated counties in other United States (U. S.) states are witnessing ∼2.4% per year or greater rates of maize yield gains. Similar doubling rates in maize yields are found in the states of Chihuahua, coastal Sinaloa, most of Michoacán and Guanajuato and isolated areas of few other Mexican states as well as El Salvador. Maize yields are decreasing in parts of the U. S. Great Plains states (southern South Dakota, Kansas, Eastern Colorado and parts of southeastern Texas), eastern Mexico (San Luis Potosí, northern Durango, southeastern Coahuila, Nuevo León and Tamaulipas), and in Haiti and Guatemala (Figure 2a). The resultant impact is that the United States has the highest national rates of maize yield improvement in this region of the world (in kg/ha/year^2^) followed by Canada, then Cuba, and Mexico (Data S1). In Central American countries such as Honduras and Nicaragua, where maize now provides ∼27%, and ∼25% of daily dietary energy, respectively [31], and in Panama (∼7% of dietary energy), the production gains from their slower 0.5% per year yield improvement rates could be less than those required to keep pace with their population growth (in per capita harvested production terms). In Guatemala, where maize now provides ∼36% of dietary energy [31] the yield trends are already negative (−0.7% per year), and as the population is projected to substantially increase [5], a steeper fall in the per capita harvested maize could occur.

Rice yield doubling rates are found only in some isolated areas of North and Central America. The United States has the highest overall rice yield improvement rates (1.2% per year), followed by Mexico (1.1% per year). In Nicaragua and Panama where rice supplies ∼16% and ∼24% of dietary energy respectively, the per capita rice harvests could fall due to their population growth [5] outpacing their 0.9% and 0.2% per year rice yield improvement rates (Figure 2b). Elsewhere in the Dominican Republic, Costa Rica, and Haiti, where rice provides 16–22% of their daily dietary energy, yields are declining at rates of −0.1% to −0.6% per year. The per capita rice production is likely to increase only in Cuba, where rice yields are increasing 0.9% per year [31] and the population is projected to fall [5].

Wheat yields are increasing at ∼2.4% per year or greater only in some counties in the U. S., (mainly in eastern South Dakota, parts of Nebraska, northeastern Kansas, western Mississippi, and Louisiana) (Figure 2c). Wheat yields are decreasing in many parts of the U. S. Great Plains (Montana, western parts of North Dakota, South Dakota, Kansas and Texas and eastern Colorado). In Mexico, areas with doubling rates in wheat yields are observed only in the state of Zacatecas. Nationally, wheat yields in Canada, United States, and Mexico are increasing at 1.3%, 0.8% and 1.1% per year, respectively.

Most areas in the U. S. show increasing soybean yields, with doubling rates in North Dakota, isolated areas of South Dakota, Nebraska, Mississippi, Louisiana, and Georgia (Figure 2d). Soybean yields are decreasing in Kansas, Oklahoma and Texas. Canada and the United States have yields increasing at 0.2% and 1.2% per year, respectively.

### South America

Most maize areas in South America are achieving doubling rates, with the exception of isolated municípios in Brazil. The overall impact of these varied subnational rates is that maize yields are increasing at 1.7–4% per year in Uruguay, Argentina, Chile, and Brazil and may result in significantly higher per capita maize harvests. Other South American countries such as Venezuela, Peru, Bolivia and Ecuador, where maize provides 2–14% of dietary energy, are achieving yield increases of 1.8–3% per year but due to their population growth [5] may result in no significant changes to their per capita maize harvests at least in the short term.

Rice is grown throughout in South America and yields are improving in most areas (Figure 2b). Rice yield rates are at doubling levels however only in the Cesar and Tolima departments of Colombia, and isolated municípios especially in the states of Pará, Maranhão, and Mato Grosso in Brazil and some areas of Paraguay, Uruguay and Argentina. Decreasing rates of rice yield are found in northeastern Brazil. The overall impact of these subnational rates of rice yield changes is: national rice yields are improving fast in Brazil, Peru, Colombia, Uruguay, and Argentina (1.6–2.7% per year). But due to population growth [5], yield increases alone may be unable to boost the per capita rice harvested in Peru, Argentina and Ecuador in the short term (∼2025). Elsewhere in Venezuela and Bolivia (where rice supplies 8–19% of daily dietary energy and rice yields are improving at 1.1–1.4% per year) per capita harvested rice could remain unchanged to 2050. In Suriname, where rice provides ∼25% of dietary energy, the very low rates of rice yield improvement, 0.2% per year, may even lead to decreased per capita rice harvests. In Brazil, Uruguay, Guyana, and Paraguay where rice now supplies ∼11%, ∼7%, ∼29%, and ∼2% of dietary energy respectively, the per capita rice harvested could increase.

Large extents of doubling wheat yield rates occur only in Argentina and Chile. Wheat yields are decreasing in parts of Peru and in Santiago del Estero in Argentina. The national trends as a consequence: Argentine and Chilean wheat yields are increasing at 1.5% and 1.9% per year respectively and may result in increased per capita wheat harvests (wheat provided ∼25% and ∼30% of dietary energy, respectively). In Brazil, Colombia, Ecuador, and Uruguay, yields are increasing at 0.1–1.5% per year and may lead to unchanged per capita harvests, (8–31% of dietary energy is now supplied from wheat in these four countries). Per capita wheat harvests could decrease in: Bolivia, Peru, and Paraguay due to lower yield gains of 0.5–1.6% per year.

With the exception of Bolivia and Paraguay, soybean yields are increasing at doubling rates, particularly in many areas of Argentina, Brazil, Venezuela, and Uruguay. Soybean yields are increasing 1.5–2.4% per year in these countries.

### Europe

Almost everywhere in Europe, except in Moldova, maize yields are increasing but rates of ∼2.4% per year are found only in Portugal, the Czech Republic, and Belarus. In Moldova, southern Romania (counties in south and southwest region) and Pomeranian province in Poland maize yields are deceasing and have led national maize yields to change at −4.9% per year in Moldova, 0.7% per year in Romania, and 1.1% per year in Poland. Due to rising maize yields of 0.8–3.0% per year, the per capita harvested maize could increase in many European countries by 2050.

Rice is harvested in only a few European countries and yields are increasing at 0.2–1.5% per year. See Data S1 for the numbers.

Wheat is an important food crop in Europe and harvested in almost all European countries. However, in Eastern Europe (Ukraine, Moldova, southern Romania, Bulgaria, parts of Hungary and Slovak Republic), southern France, and northeastern Spain wheat yields are generally decreasing with the exception of a few regions where yield increases are at doubling rates. Consequently, national wheat yield improvements in European countries are generally <1% per year, with the exception of Estonia (1.5% per year). Even though wheat yield improvements are low, the per capita harvested wheat may increase in some of these European countries because of population declines in Estonia, Germany, Latvia, and Lithuania [5]. Unfortunately, in many other European countries, the low production gains from yield improvement will likely be offset by increasing population, resulting in nearly unchanged per capita wheat harvests. Yields are decreasing in many eastern European countries throughout, where wheat comprises 24–36% of the dietary energy.

Soybean yields are increasing at doubling rates only in small areas in Romania, and in central Italy soybean yields are decreasing. See Data S1 for actual national numbers.

### Africa

Africa is a continent of contrasts with regards to rates of maize yield change. For example, maize yields are increasing ∼2.4% per year in the Nigerian states of Yobe and Adamawa. Similar maize yield improvement rates are found in some other isolated areas of West African nations, Ethiopia, Angola, South Africa, and Madagascar. But maize yields are decreasing in Morocco, Chad, Somalia, Kenya, Zambia, Zimbabwe, Rwanda, Burundi, and Democratic Republic of Congo. Elsewhere, rates of yield improvement are lower than population growth, suggesting that production per capita is likely to decline. These trends are particularly troubling in countries such as Burundi, Chad, Kenya, Morocco, Rwanda, Democratic Republic of Congo, Somalia, Zambia, and Zimbabwe, where yields are decreasing −0.2% to −7.6% per year, population is rising [5], and maize accounts for 5–51% of calorie intake. The only African countries that may witness an increase in per capita maize harvests due to faster maize yield increases are Angola, Ivory Coast, and Mozambique, where yields are increasing at rates of 2.9%, 4.1%, and 3.2% per year, respectively.

In Ivory Coast, Togo and Benin in West Africa, and in Rwanda, rice yield changes are at doubling rates. In contrast, yields are decreasing more than 1% per year in Gambia and 3% per year in Nigeria. Nearly 8% of the dietary energy in Nigeria is supplied from rice [31]. The per capita rice harvests could decrease in almost all the important rice consuming African nations, e.g., Guinea, Madagascar, Mali, Nigeria, and Tanzania, unless yields are boosted further. Only in Ivory Coast there could be an increase on account of its ∼2.6% per year yield increases.

Wheat, while grown in only an extremely small area of Africa, though in many countries, is generally increasing yields at high rates. In Angola, Eritrea Malawi, Nigeria, Algeria, Sudan, and South Africa, yields are growing at doubling rates (2.4–3.4% per year).

In Nigeria, and Mpumalanga province of South Africa soybean yields are increasing at doubling rates whereas in Zimbabwe, Democratic Republic of Congo, and Rwanda yields are decreasing.

### Asia and Australasia

Maize yields are generally increasing across Asia. Yield improvement rates are currently on track to double production in some parts of Iran, Pakistan, India, China, Indonesia, Bangladesh, Laos, Cambodia, Vietnam, and Turkey (Figure 2a). In China, Laos, Philippines, Australia, India, Pakistan, and in Turkey, the per capita maize harvested could remain unchanged in the short term but could increase by 2050. In a few countries such as Nepal, Kyrgyzstan, Iraq, Afghanistan, Uzbekistan, and in New Zealand the slower rates of maize yield improvements of 1.3%, 0.3%, 0.9%, 1.1%, 1.6%, and 0.5% per year respectively can result in declines in per capita maize harvests on account of population growth [5]. The per capita maize harvests could increase in Indonesia, Vietnam, Thailand Iran, Myanmar, Bangladesh, Cambodia, Azerbaijan, and in Bhutan [5].

Rice areas with doubling yield rates are found only in some local areas within Afghanistan, India, Bangladesh, Laos, Vietnam and Cambodia. Significant rates of rice yield declines are found in parts of India (especially in parts of Uttar Pradesh, Maharashtra, and Tamil Nadu) and in North Korea. The ∼1% per year overall rice yield increase in India could result in no significant change to the overall per capita rice harvested but in China this may remain as only a short term (∼2025) problem. Rice provides ∼30% and ∼27% of the dietary energy in India and China respectively now. On the other hand in the world's third largest rice producer, Indonesia where ∼49% of dietary energy is provided by rice, yield improvement rates are much lower at 0.4% per year. The other important Asian rice producers may behave in the following way: no significant change in per capita rice harvests in Pakistan, Nepal, Malaysia, and South Korea; no significant increase in Myanmar, Sri Lanka, Turkey and Bhutan only in the short term, and declines in the Philippines in the long term (∼2050). However in Afghanistan, Iraq and Australia due to population growth outpacing production increases from the 2.4%, 0.4%, and 0.3% rates of yield increase per year there could be declines in per capita harvested rice. In North Korea, Kazakhstan, Uzbekistan, and in Turkmenistan yields are declining at −2%, −1.9%, −0.3%, and −1.5% per year respectively. Elsewhere, the per capita rice harvested could increase: Bangladesh, Vietnam, Thailand, Cambodia, Laos, and in Iran due to production increases from high rates of yield improvement (1–2.6% per year) outpacing their population growth [5], and in Japan due to small yield improvement rates (0.5% per year) and small population decreases.

Wheat yields are increasing at doubling rates in parts of Iraq, Iran, and Afghanistan, but only in small parts of the top producing countries of China and India. Wheat yields are decreasing in many areas of India, especially in the states of Madhya Pradesh and Uttaranchal, and in the countries of Kyrgyzstan, and Mongolia, and in the Beijing province of China. Large areas with wheat yield decreases are also found in Queensland, New South Wales, Victoria, and Western Australia, in Australia. The consequence of these wheat yield change rates is diverse. Per capita wheat production could increase in many countries, including China, Iran, and North Korea, because yield increases exceed projected population increases. In contrast, decreases in per capita harvests could occur in: Afghanistan, Georgia, Iraq, Kyrgyzstan, Mongolia, Saudi Arabia, Yemen, and in Australia.

Soybean yields are increasing at ∼2.4% throughout China, including the provinces of Jilin, Guangxi, and Guangdong, but are decreasing in Yunnan and Ningxia. Yields are increasing at doubling rates in local areas within Maharashtra in India. In contrast, yields are generally decreasing in the neighboring state of Madhya Pradesh. Doubling rates of soybean yields are also found in Laos and Vietnam, but in North Korea, and Cambodia, yields are decreasing.

## Discussion and Conclusions

Numerous studies have shown that feeding a more populated and more prosperous world will roughly require a doubling of agricultural production by 2050 [1]–[7], translating to a ∼2.4% rate of crop production growth per year. We find that the top four global crops – maize, rice, wheat, and soybean – are currently witnessing average yield improvements only between 0.9 to 1.6 percent per year, far slower than the required rates to double their production by 2050 solely from yield gains. This is because yield improvements are below ∼2.4% per year in many areas of our most important agricultural lands. At these rates maize, rice, wheat and soybean production may increase by ∼67%, ∼42%, ∼38%, and ∼55% respectively, by 2050 globally. There is a 90% chance that the total global production increase from yields alone would be between 34–101% for maize, 21–59% for rice, 4–76% for wheat, and 13–84% for soybean by ∼2050. Thus, if these yield change rates do not increase, land clearing possibly would be needed [3] if global food security is to increase or even maintained (Table 1).

We found that the top three rice and wheat producing nations are witnessing very low yield growth rates. China, India and Indonesia are witnessing rice yield increases of only 0.7%, 1.0%, and 0.4% improvement per year. China, India, and the U. S., the top three wheat producers similarly were witnessing yield increases of only 1.7%, 1.1%, and 0.8% per year, respectively. At these rates we found that yield driven production growth in India and China could result in nearly unchanged per capita rice harvests, but decline steeply in Indonesia.

In many of the smaller crop producing nations, maize, rice, or wheat yield improvement rates are below the 2.4% doubling rate. Unfortunately, a high percentage of total calories consumed in these countries are from these four crops. This is particularly true for maize throughout much of Africa (e.g., Kenya, Zambia, Zimbabwe), Central America (e.g., Guatemala, Nicaragua, Panama), and parts of Asia (e.g., Nepal, Georgia).

Rice provides ∼19% of dietary energy globally. Rice provides a higher percentage of total calories consumed in countries such as Dominican Republic, Costa Rica, Haiti, Sierra Leone, Nigeria, and North Korea, yet yields are declining, −0.1% to −3.2% per year. Elsewhere rice yields are increasing too slowly to overcome the impact of their population growth. In some of the world's top rice producers, e.g. India and China, the per capita production may remain nearly unchanged. In numerous smaller rice producers across the world where rice is an important significant provider of daily dietary energy such as in Peru, Ecuador, Bolivia, Benin, Togo, Myanmar, Philippines, Malaysia, South Korea, Nepal, and in Sri Lanka, the per capita production may also remain unchanged.

Wheat provides ∼19% of global dietary energy. Wheat comprises an even larger portion of the diet in some countries where yields are declining, particularly Eastern European countries of Bulgaria, Hungary, Czech Republic, Moldova, Romania, Slovakia, and Ukraine. In many countries, such as Bolivia, Peru, Paraguay, Afghanistan, and Iraq, wheat yield increases are too low to maintain their current per capita harvests.

Our analysis identifies where yield improvements are on track to double production and where investments should be targeted to increase yields. The observed rates of yield change result from several location-specific, socio-economic, and biophysical factors that are described elsewhere [23]. Many studies illustrate that intensification can be unsustainable [32]–[36], but several notable projects in Africa [37] and elsewhere [38] have shown that sustainable intensification is possible and necessary to boost global crop production.

Clearly, the world faces a looming and growing agricultural crisis. Yields are not improving fast enough to keep up with projected demands in 2050. However, opportunities do exist to increase production through more efficient use of current arable lands [4] and increasing yield growth rates by spreading best management practices and closing yield gaps under different management regimes [38]–[42] across the globe. A portion of the production shortfall could also be met by expanding croplands, but at a high environmental cost to biodiversity and carbon emissions [4], [43]–[45]. Alternatively, additional strategies, particularly changing to more plant-based diets and reducing food waste [4], [46]–[48] can reduce the large expected demand growth in food [3], [4].

## Methods

### Data

We used annual crop census reports for harvested areas and yield from ∼13,500 political units globally covering 20 years from 1989 to 2008 in this analysis though the database itself covers the years 1961 to 2008. The sum total of these census reports for the 20 years was approximately 1.8 million. Data were collected at three political levels/units depending on data availability: country, state/provinces, and county/district/município/department. Data were not available for all political units for each year. Details of the number of years data was available and its source is given in the Table S1. For the political units where data was missing for some years we estimated crop harvested and yield information using the average of the latest five years of reported data and constraining them with the reported numbers from the higher political unit as explained further in Text S1 and previous work [23].

Population data and its projections per country were from the United Nation's medium variant projections [5]. Crop production was determined using the projected crop yields at current observed rates of yield change and harvested areas fixed at ∼2007. Per capita harvested production is the ratio of production to population and a greater than ±10% change from ∼2007 is considered as significant either in the short- (2025) or long-term (2050).

### Analysis

We linearly regressed 20 years of crop yields at each of the political units to determine the average linear rates of yield improvement over the observed period. Many previous studies have shown that crop yields change linearly and have used linear regression to project future crop yields [24]–[30]. Here we calculate the non-compounding linear percentage rate by solving *a* in Equation 1; *Y* is the yield in the year 2008, *2Y* is the yield in 2050 (after *42* years):
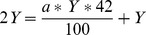
(1)This gives a rate of 2.38% per year or approximately 2.4% per year. For reported numbers at the local- to country- to global-scale the linear percentage changes are the observed changes using 2008 yields as the base year. The actual changes are provided in Data S1 for each crop and country.

Details of the method used, sensitivity to the number of years analyzed, as well as alternate regression methods are provided in Figures S6 and S9. The advantage of analyzing at high spatial resolution is that yield rates can be summarized for other unique levels. For example, we summarized the results for the Brazilian Legal Amazon (Figure S12 and Table S2). We compared our global numbers with other reported estimates. These comparisons are provided in Table S3.

## Supporting Information

Figure S1 Global maps of the coefficient of variation (r^2^) for maize, rice, wheat, and soybean when fitted to 20 years of yield information at each political unit analyzed.
(TIFF)

Figure S2 Global maps of normality of the data for maize, rice, wheat, and soybean at each political unit analyzed from the Lilliefors test (green colors show where the normality assumptions are not violated at p>0.05 and red colors where they are violated at p≤0.05).
(TIFF)

Figure S3 Global maps of autocorrelation of the data for maize, rice, wheat, and soybean at each political unit analyzed from the Durbin-Watson test (green colors show where the autocorrelation assumptions are violated at p>0.05 and red colors where they hold at p≤0.05).
(TIFF)

Figure S4 Diagnostic plots for a linear fit (r^2^ = 0.49, p<0.01) to soybean yield data in the United States. Subplots show a) model fit and standard 95% confidence interval, b) QQ plot, c) residuals versus fitted values, and d) residuals versus lagged residuals. Durbin-Watson test for autocorrelation: p = 0.66. Lilliefors test for normality of yield data: p>0.5.
(TIFF)

Figure S5 Diagnostic plots for a linear fit (r^2^ = 0.49, p<0.01) to maize yield data in Angola. Subplots show a) model fit and standard 95% confidence interval, b) QQ plot, c) residuals versus fitted values, and d) residuals versus lagged residuals. Durbin-Watson test for autocorrelation: p<0.05. Lilliefors test for normality of yield data: p>0.5.
(TIFF)

Figure S6 Parsimoniously fitted yields at each of the political units and using them to project global crop yields to the year 2025.
(TIFF)

Figure S7 Diagnostic plots for a quadratic fit (r^2^ = 0.60, p<0.01) to maize yield data in Angola. Subplots show a) model fit and standard 95% confidence interval, b) QQ plot, c) residuals versus fitted values, and d) residuals versus lagged residuals. Durbin-Watson test for autocorrelation: p = 0.08.
(TIFF)

Figure S8 Consequences of extrapolating linear and quadratic maize yield models for Angola to 2050.
(TIFF)

Figure S9 Global maize, rice, wheat, and soybean yield fitted to 15 and 25 years of data and using it to project yields to the year 2025. Dashed lines correspond to analysis using 15 years of data (1994–2008), dotted lines correspond to using 25 years of data (1984–2008), and solid lines correspond to using 20 years of data (1989–2008). Due to the similarity in results in some cases all lines are not clearly distinguishable from each other always.
(TIFF)

Figure S10 Rates of yield change in kg/ha/year/year.
(TIFF)

Figure S11 Year 2008 yields.
(TIFF)

Figure S12 Similar to Figure 1 in the main text but only for the Brazilian Legal Amazon.
(TIFF)

Table S1 Country data source, number of political units analyzed per country, time frame and number of official statistics collected per crop for the period 1989 to 2008.
(DOCX)

Table S2 Current yields, projections and production for the Brazilian Legal Amazon.
(DOCX)

Table S3 Comparison with the future U. S. crop yields reported by the USDA-ERS [91] (maize, wheat, and soybean were reported in bushels per acre, rice in pounds per acres from USDA-ERS and converted to ton/ha), and global wheat yields reported by the FAO-OECD [90].
(DOCX)

Text S1 Additional Data, Model Fitting, Rates, and Inter-comparison information.
(DOC)

Data S1 Rates observed for each crop and country.
(XLSX)
