# Supplementary figures and images for: Yield Trends Are Insufficient to Double Global Crop Production by 2050
Source: PLoS One. 2013 Jun 19;8(6):e66428. doi: 10.1371/journal.pone.0066428 (PMC3686737; doi:10.1371/journal.pone.0066428)

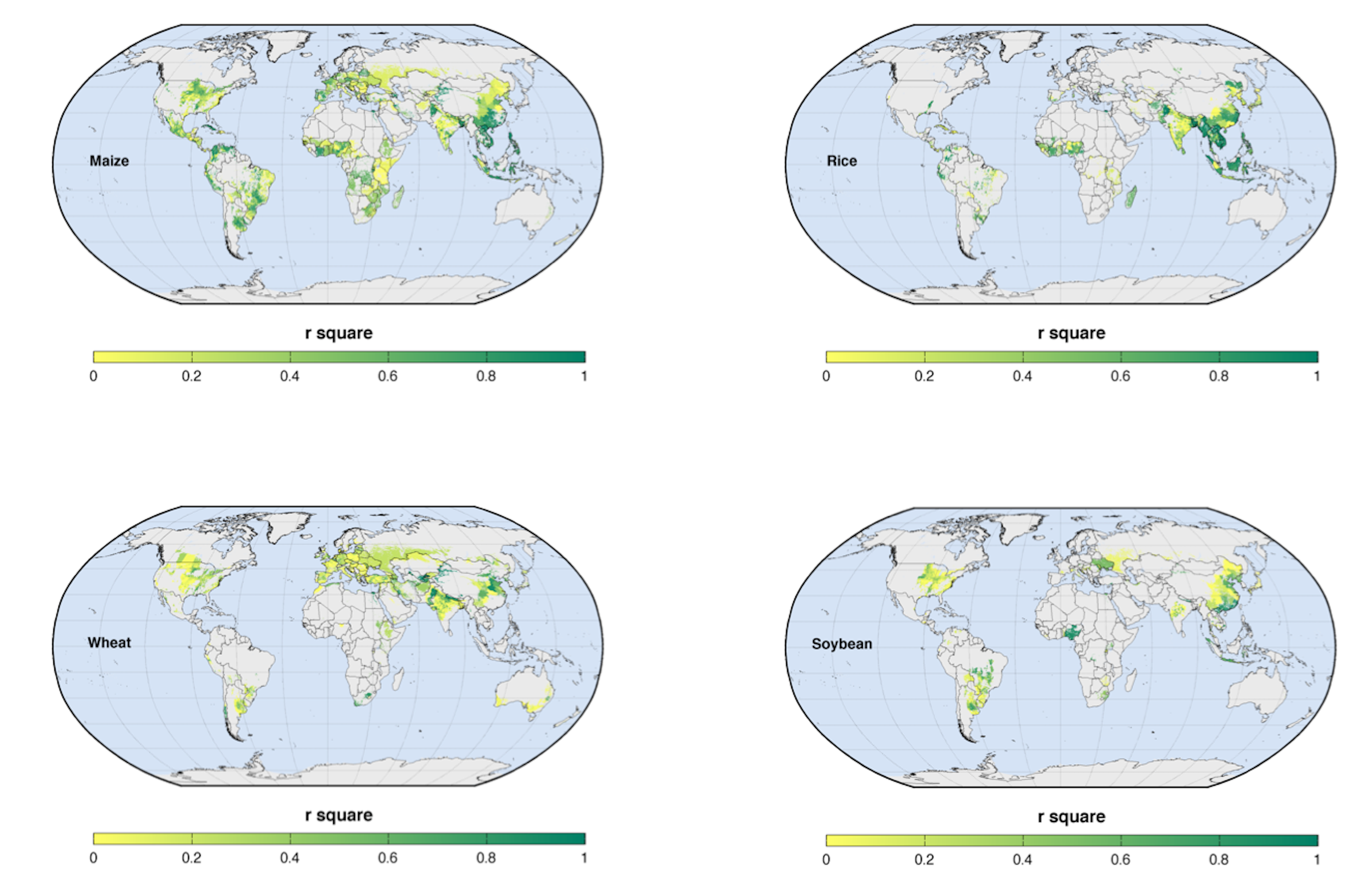

Supplement: Figure S1 — Global maps of the coefficient of variation (r2) for maize, rice, wheat, and soybean when fitted to 20 years of yield information at each political unit analyzed. (TIFF) [file pone.0066428.s001.tiff]

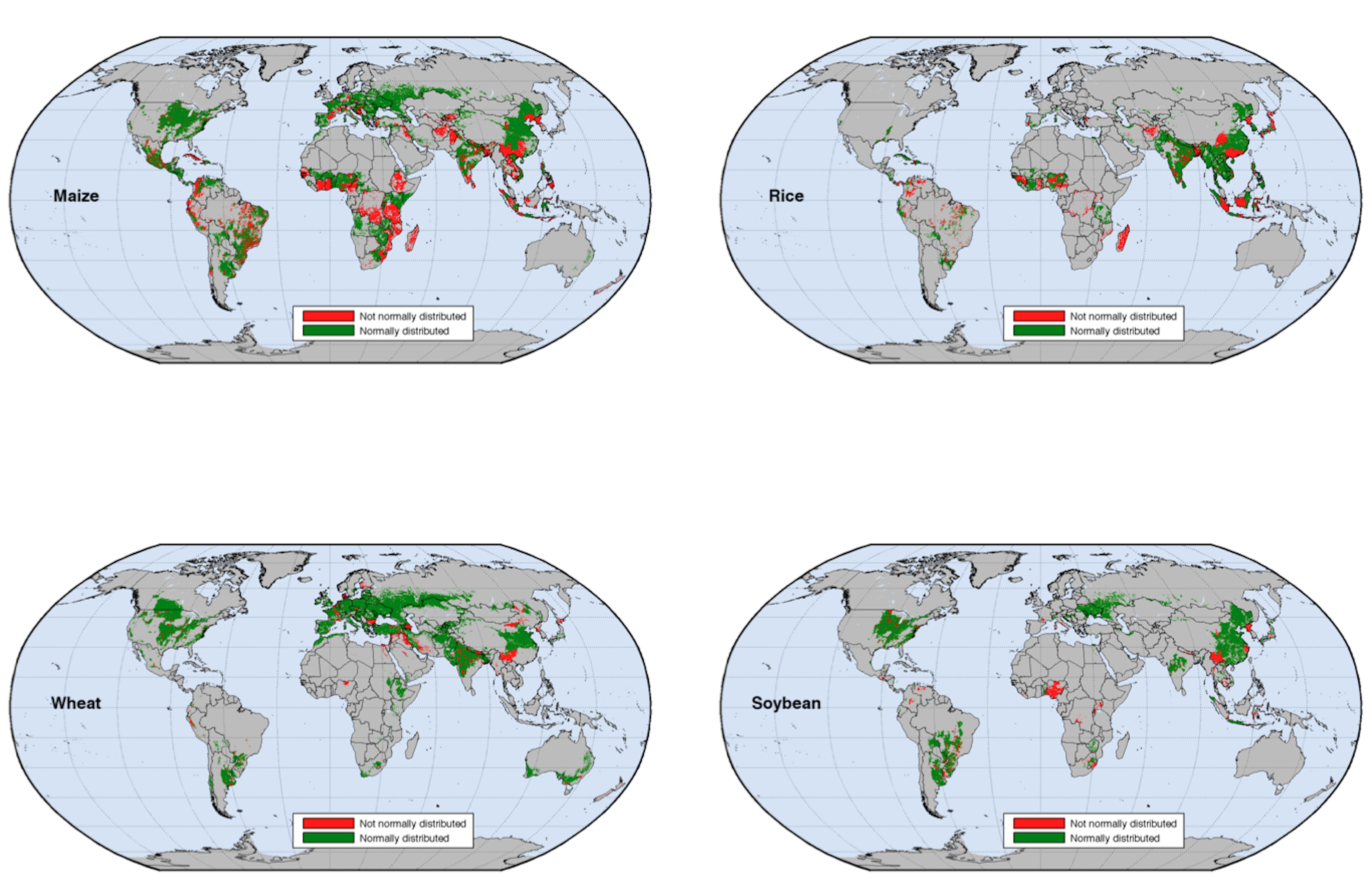

Supplement: Figure S2 — Global maps of normality of the data for maize, rice, wheat, and soybean at each political unit analyzed from the Lilliefors test (green colors show where the normality assumptions are not violated at p>0.05 and red colors where they are violated at p≤0.05). (TIFF) [file pone.0066428.s002.tiff]

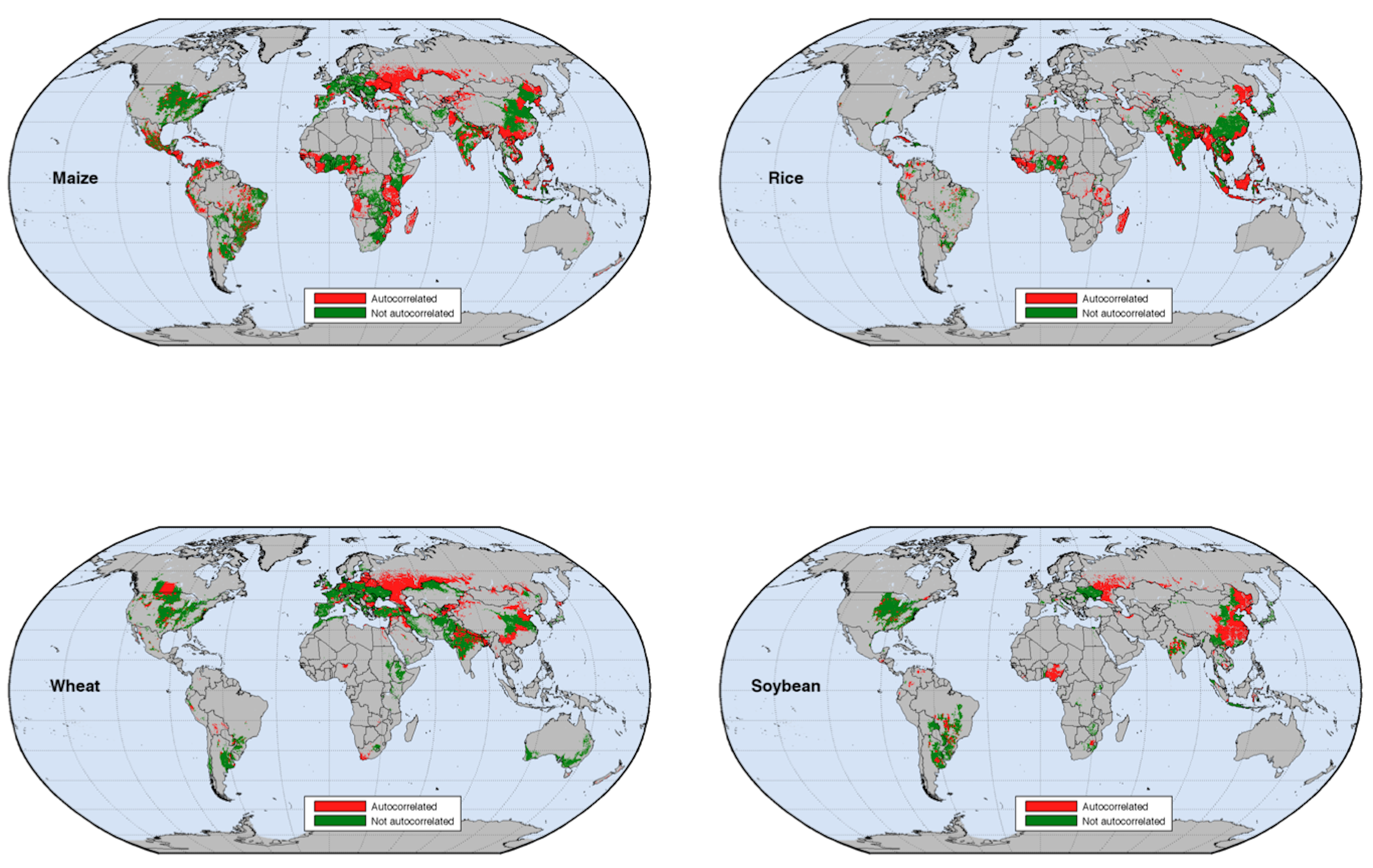

Supplement: Figure S3 — Global maps of autocorrelation of the data for maize, rice, wheat, and soybean at each political unit analyzed from the Durbin-Watson test (green colors show where the autocorrelation assumptions are violated at p>0.05 and red colors where they hold at p≤0.05). (TIFF) [file pone.0066428.s003.tiff]

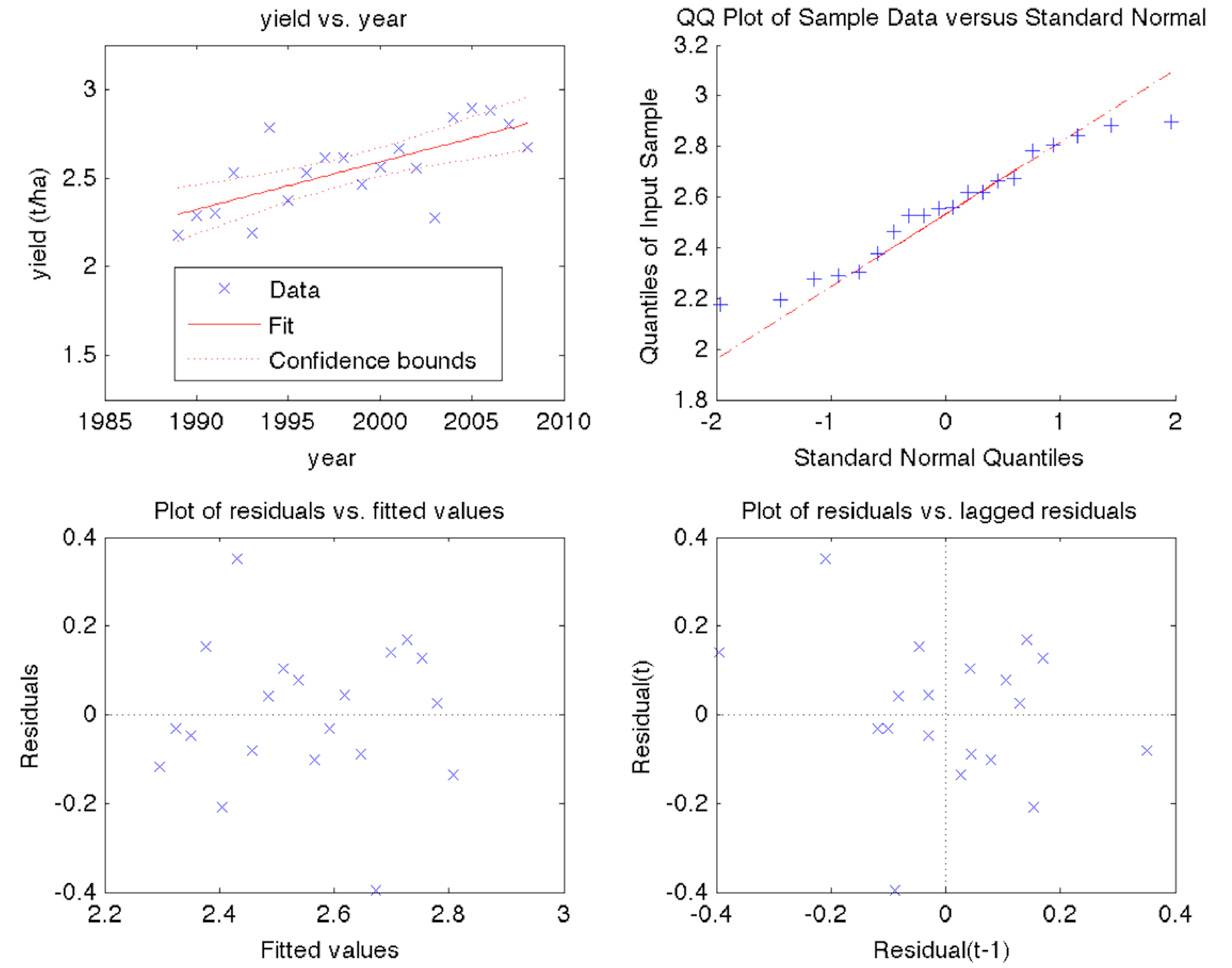

Supplement: Figure S4 — Diagnostic plots for a linear fit (r2 = 0.49, p<0.01) to soybean yield data in the United States. Subplots show a) model fit and standard 95% confidence interval, b) QQ plot, c) residuals versus fitted values, and d) residuals versus lagged residuals. Durbin-Watson test for autocorrelation: p = 0.66. Lilliefors test for normality of yield data: p>0.5. (TIFF) [file pone.0066428.s004.tiff]

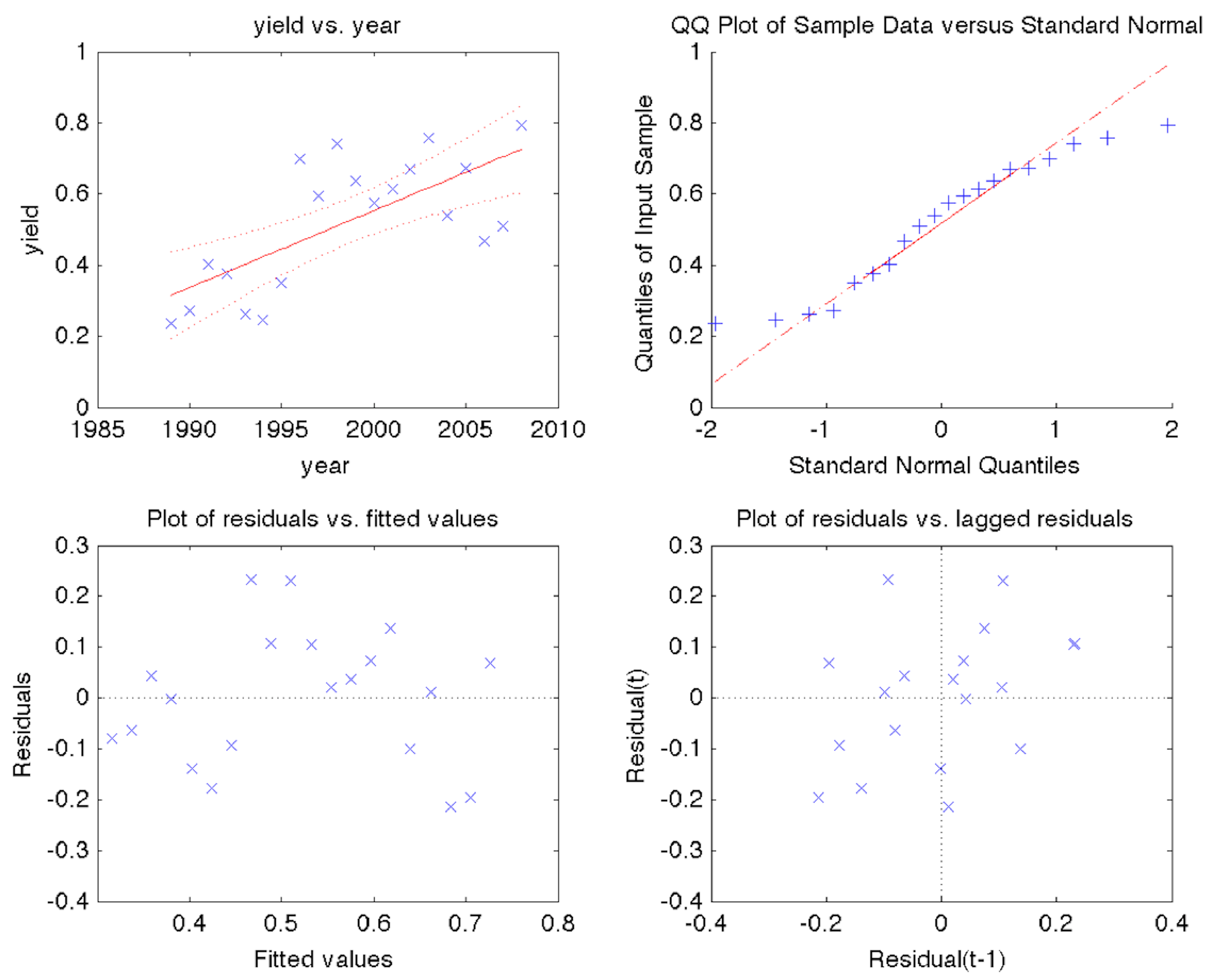

Supplement: Figure S5 — Diagnostic plots for a linear fit (r2 = 0.49, p<0.01) to maize yield data in Angola. Subplots show a) model fit and standard 95% confidence interval, b) QQ plot, c) residuals versus fitted values, and d) residuals versus lagged residuals. Durbin-Watson test for autocorrelation: p<0.05. Lilliefors test for normality of yield data: p>0.5. (TIFF) [file pone.0066428.s005.tiff]

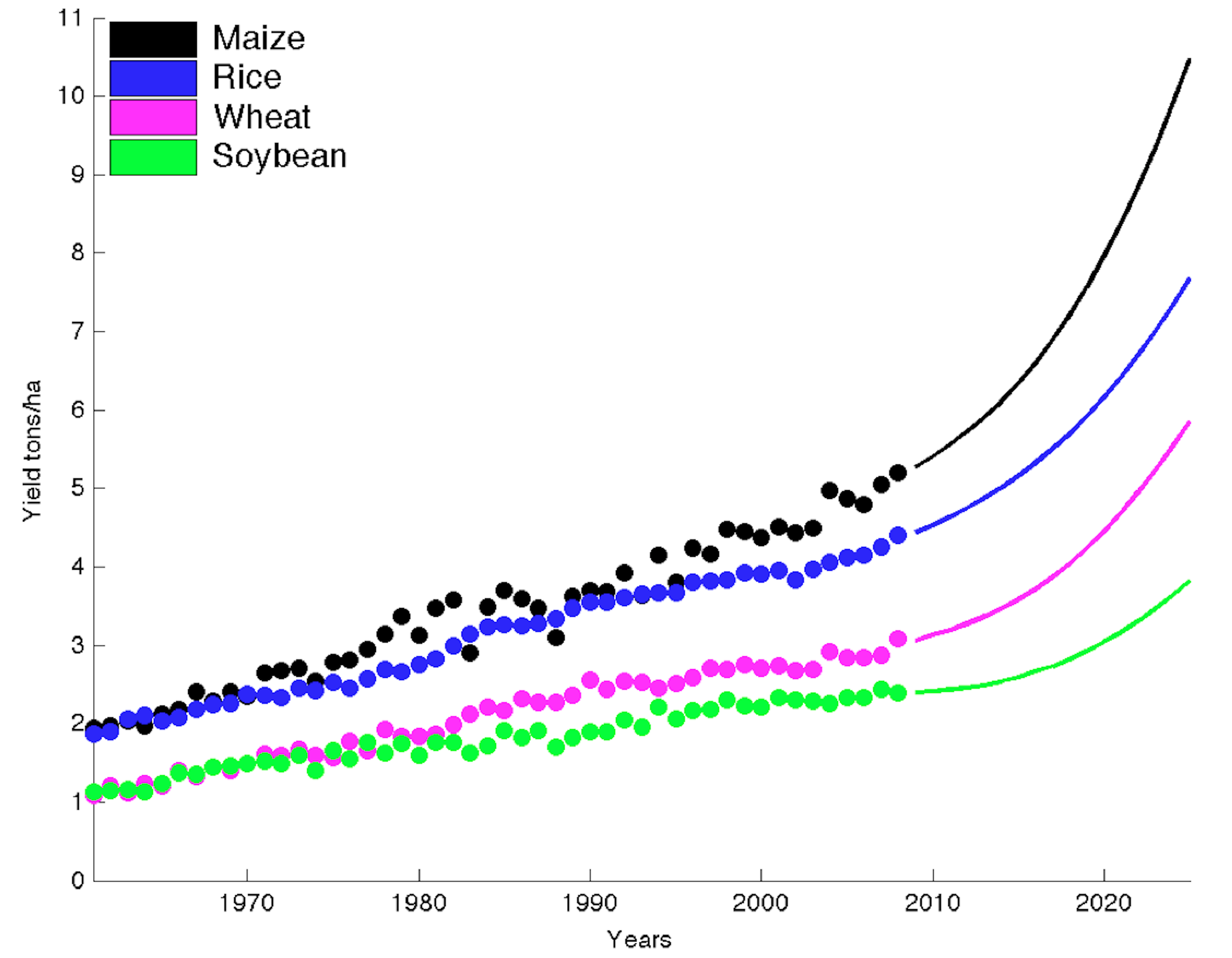

Supplement: Figure S6 — Parsimoniously fitted yields at each of the political units and using them to project global crop yields to the year 2025. (TIFF) [file pone.0066428.s006.tiff]

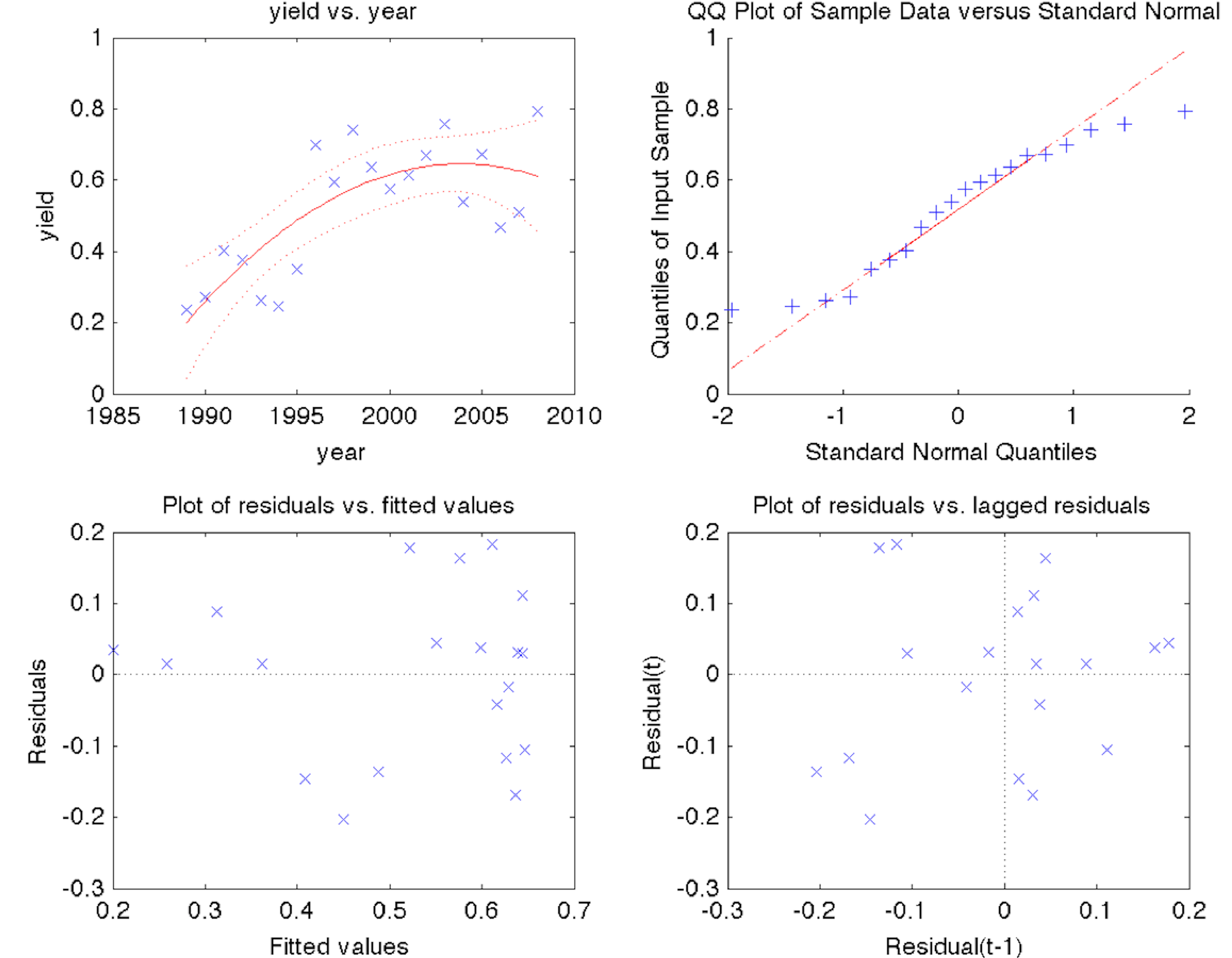

Supplement: Figure S7 — Diagnostic plots for a quadratic fit (r2 = 0.60, p<0.01) to maize yield data in Angola. Subplots show a) model fit and standard 95% confidence interval, b) QQ plot, c) residuals versus fitted values, and d) residuals versus lagged residuals. Durbin-Watson test for autocorrelation: p = 0.08. (TIFF) [file pone.0066428.s007.tiff]

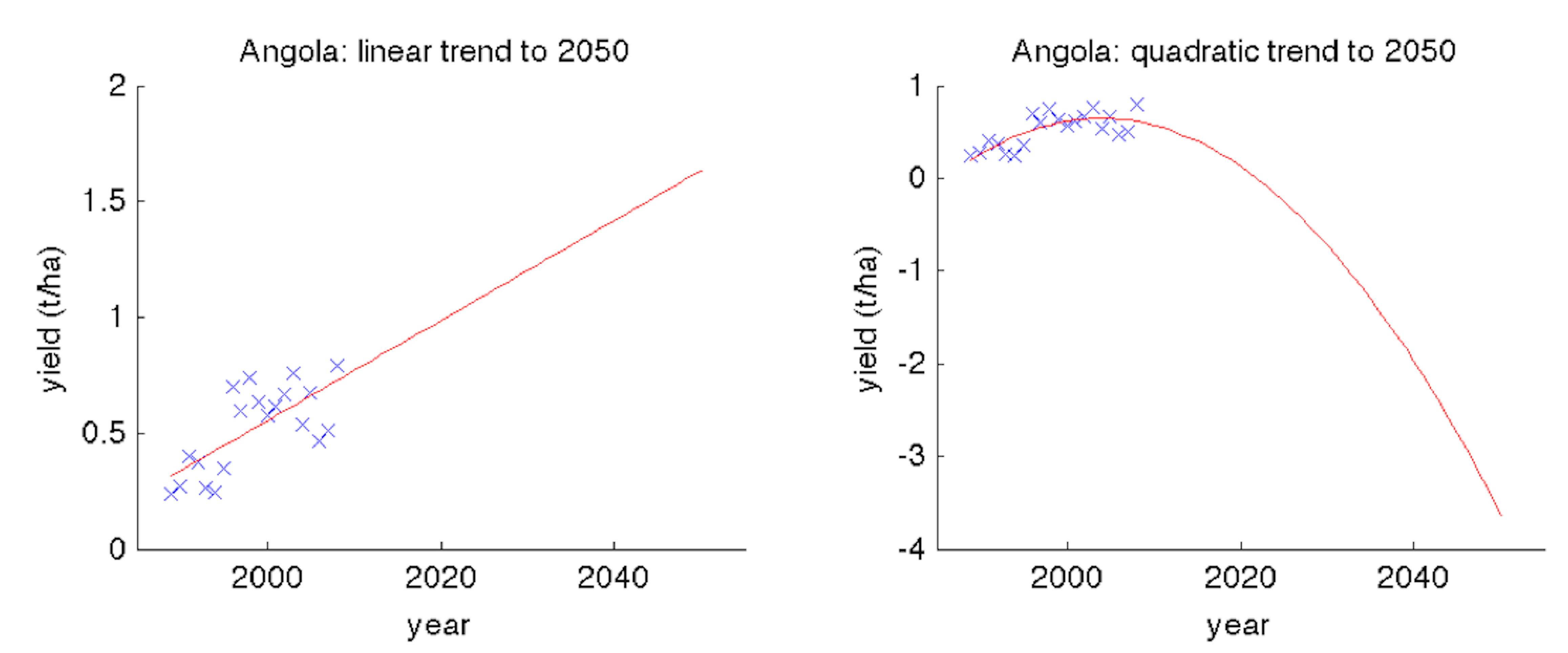

Supplement: Figure S8 — Consequences of extrapolating linear and quadratic maize yield models for Angola to 2050. (TIFF) [file pone.0066428.s008.tiff]

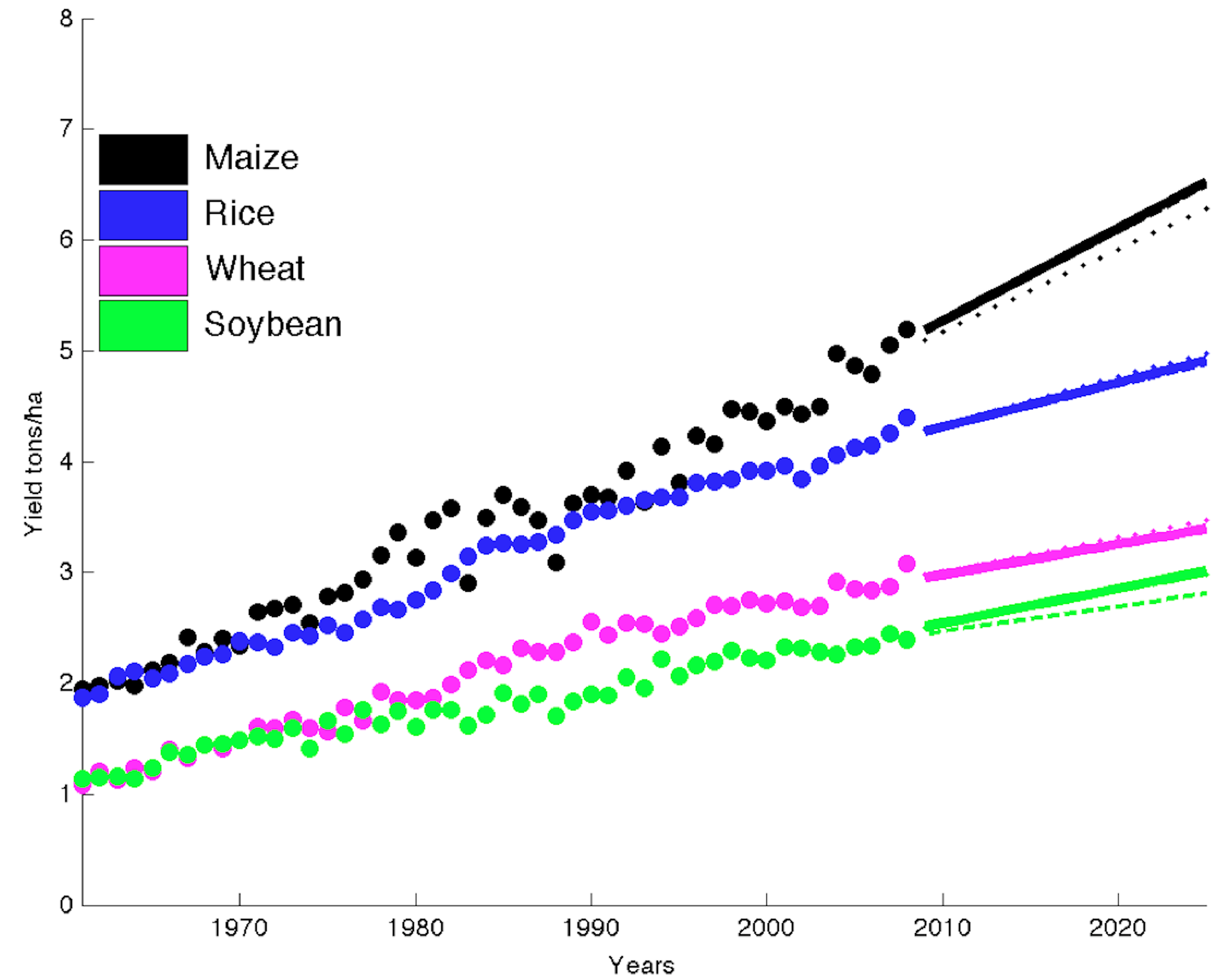

Supplement: Figure S9 — Global maize, rice, wheat, and soybean yield fitted to 15 and 25 years of data and using it to project yields to the year 2025. Dashed lines correspond to analysis using 15 years of data (1994–2008), dotted lines correspond to using 25 years of data (1984–2008), and solid lines correspond to using 20 years of data (1989–2008). Due to the similarity in results in some cases all lines are not clearly distinguishable from each other always. (TIFF) [file pone.0066428.s009.tiff]

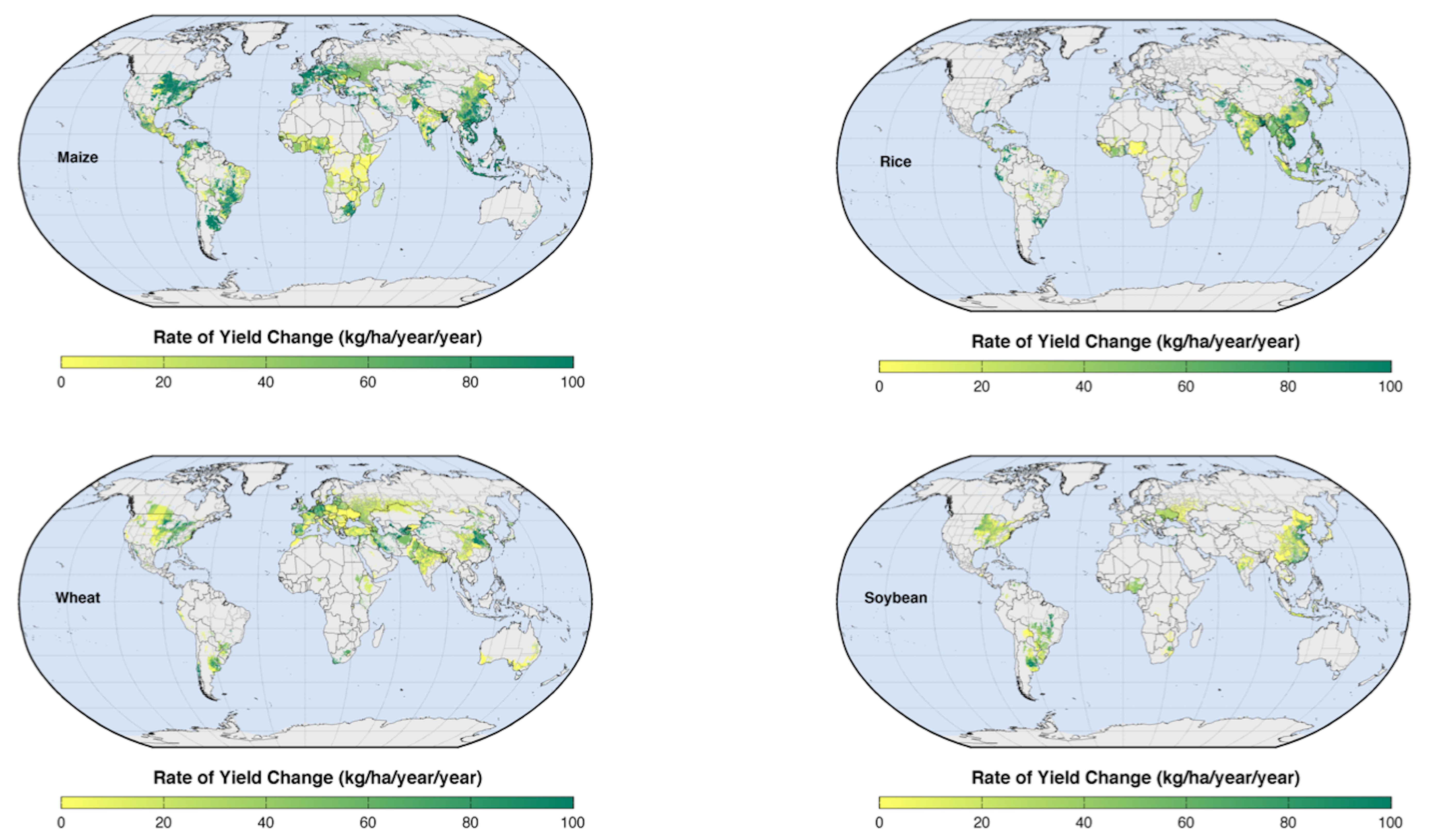

Supplement: Figure S10 — Rates of yield change in kg/ha/year/year. (TIFF) [file pone.0066428.s010.tiff]

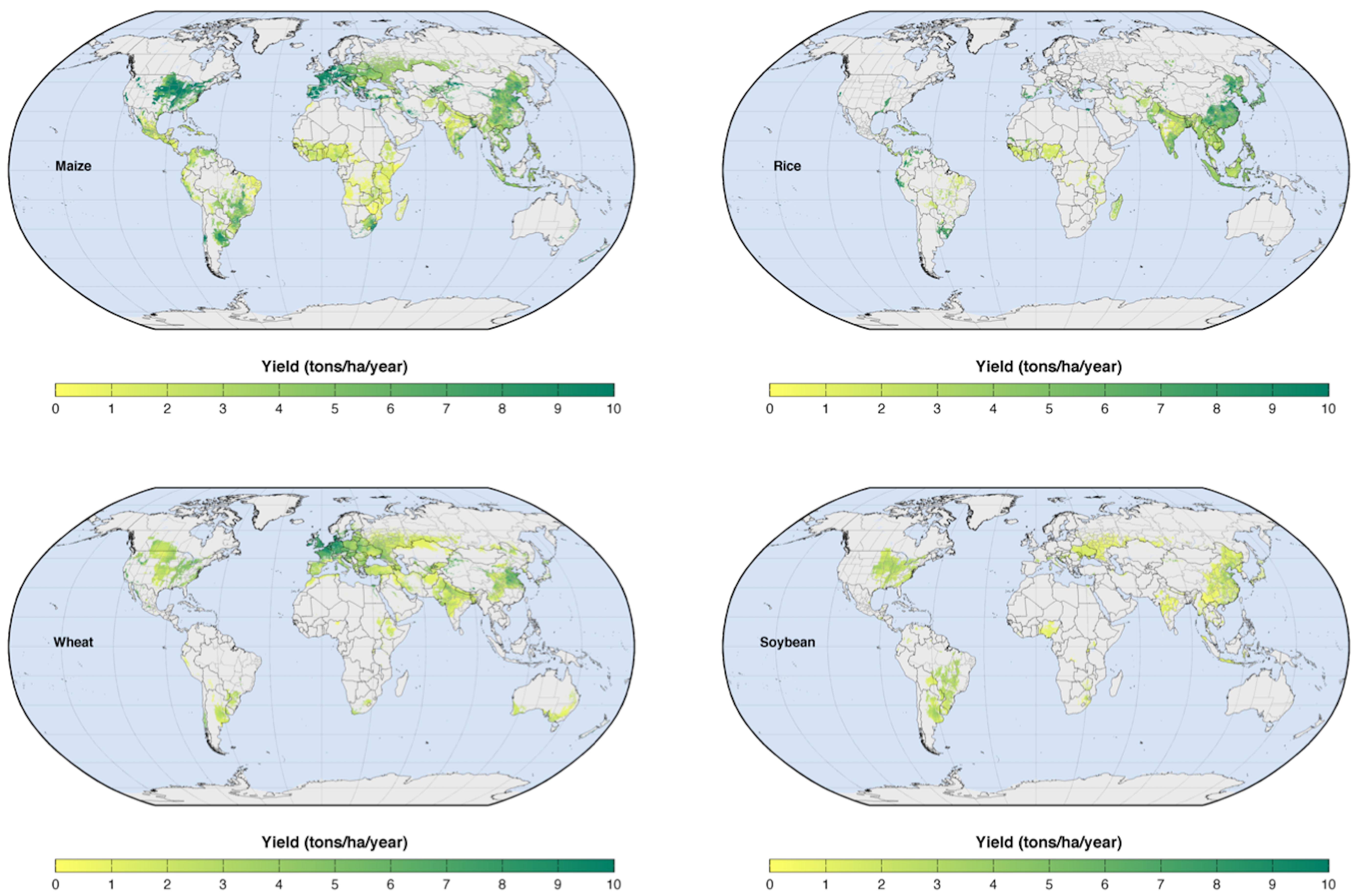

Supplement: Figure S11 — Year 2008 yields. (TIFF) [file pone.0066428.s011.tiff]

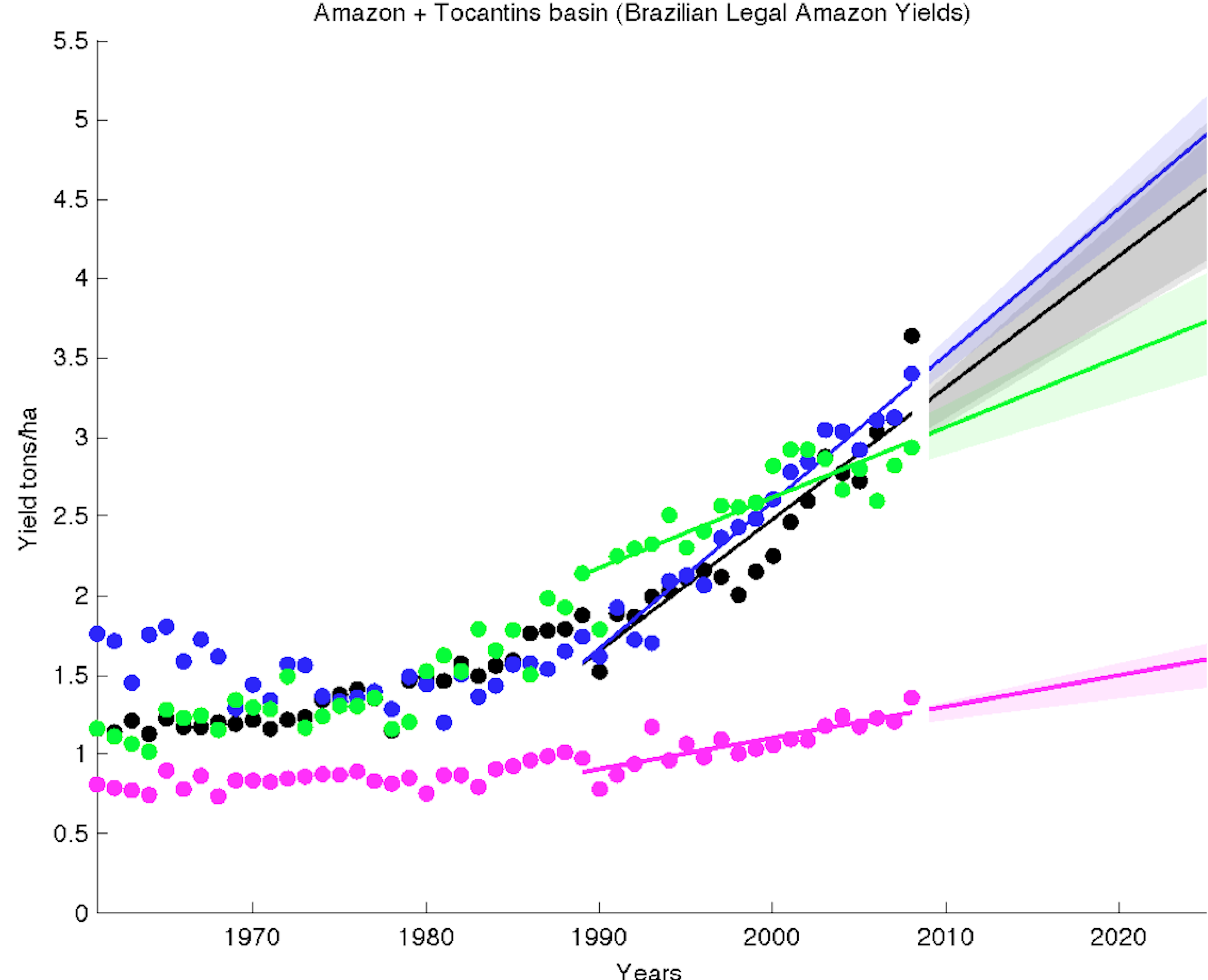

Supplement: Figure S12 — Similar to Figure 1 in the main text but only for the Brazilian Legal Amazon. (TIFF) [file pone.0066428.s012.tiff]
