# Supplementary material for: Yield Trends Are Insufficient to Double Global Crop Production by 2050
Source: PLoS One. 2013 Jun 19;8(6):e66428. doi: 10.1371/journal.pone.0066428 (PMC3686737; doi:10.1371/journal.pone.0066428)
Supplement: Table S1 — Country data source, number of political units analyzed per country, time frame and number of official statistics collected per crop for the period 1989 to 2008. (DOCX) [file pone.0066428.s013.docx]

**Ray et al. Supporting Information Table S1.** Country data source, number of political units analyzed per country, time frame and number of official statistics collected per crop for the period 1989 to 2008.

|  | Country | Source | Number of political units | Time frame (number of years available from 1989 to 2008) | Number of census reported statistics collected per crop |
| --- | --- | --- | --- | --- | --- |
| 1 | Argentina | [49], [50] | 499 municipios + 22 states + 1 national level | 20 municipios + 20 states + 20 national | 19960 municipios + 880 states + 40 national |
| 2 | Australia | [49], [51], [52], [53] | 59 statistical divisions + 9 states + national level | 10 statistical divisions + 20 states + 20 national | 1180 statistical divisions + 360 states + 40 national |
| 3 | Austria | [49], [54] | 9 states + 3 regions + national level | 20 states + 20 regions + 20 national | 360 states + 120 regions + 40 national |
| 4 | Belgium | [49], [54] | 11 states + national level (prior to 2000 we used Belgium-Luxembourg) | 20 states + 20 national | 440 states + 40 national |
| 5 | Bolivia | [49], [55] | 9 regions, + national level | 11 regions + 20 national | 198 regions + 40 national |
| 6 | Brazil | [49], [56], [57] | 5510 municipios + 27 states + national level | 20 municipios + 20 states + 20 national | 220400 municipios + 1080 states + 40 national |
| 7 | Bulgaria | [49], [54] | 28 provinces + national level | 1 provinces + 20 national | 56 provinces + 40 national |
| 8 | Canada | [49], [58] | 13 (provinces & territories) + national | 20 (provinces & territories) + 20 national | 520 (provinces & territories) + 40 national |
| 9 | Chile | [49], [59] | 13 regions + national | 20 regions + 20 national | 520 regions + 40 national |
| 10 | China | [49], [60] | 30 provinces/autonomous regions/municipalities + national | 20 provinces + 20 national | 1200 provinces + 40 national |
| 11 | Colombia | [49], [61] | 33 departments + national level | 14 departments + 20 national | 924 departments + 40 national |
| 12 | Czech Republic | [49], [54], [62] | 8 states + national level | 14 states + 20 national | 224 states + 40 national |
| 13 | Ecuador | [49], [63] | 22 states + national level | 9 states + 20 national | 396 states + 40 national |
| 14 | Finland | [49], [54] | 6 counties + 2 states + national level | 17 counties + 17 states + 20 national | 204 (counties) + 68 (states) + 40 national |
| 15 | France | [49], [54] | 22 counties + 8 states + national level | 20 counties + 20 states + 20 national | 880 (counties) + 320 (states) + 40 national |
| 16 | Germany | [49], [54] | 16 states + national | 20 states + 20 national | 640 states + 40 national |
| 17 | Greece | [49], [54] | 13 counties + 4 states + national | 20 counties + 20 states + 20 national | 520 (counties) + 160 (states) + 40 national |
| 18 | Hungary | [49], [54] | 7 states + national | 14 states + 20 national | 196 states + 40 national |
| 19 | India | [49], [64], [65], [66] | 552 districts + 32 states + national | 20 districts + 20 states + 20 national | 22080 districts + 1280 states + 40 national |
| 20 | Indonesia | [49], [67] | 26 states + national level | 16 states + 20 national | 832 states + 40 national |
| 21 | Iran | [49], [68] | 24 states + national level | 20 states + 20 national | 960 states + 40 national |
| 22 | Ireland | [49], [54] | 2 states + national level | 19 states + 20 national | 76 states + 40 national |
| 23 | Italy | [49], [54] | 20 counties + 12 states + national level | 20 counties + 20 states + 20 national | 800 counties + 480 states + 40 national |
| 24 | Japan | [49], [69] | 9 states + national level | 20 states + 20 national | 360 states + 40 national |
| 25 | South Korea | [49], [70] | 14 states + national level | 20 states + 20 national | 560 states + 40 national |
| 26 | Mexico | [49], [71] | 2402 counties + 32 states + national level | 6 counties + 20 states + 20 national | 28824 counties + 1280 states + 40 national |
| 27 | Mongolia | [49], [72] | 20 states + national level | 13 states + 20 national | 520 states + 40 national |
| 28 | Nepal | [49], [69] | 14 states + national level | 20 states + 20 national | 560 states + 40 national |
| 29 | Netherlands | [49], [54] | 12 counties + 4 states + national level | 20 counties + 20 states + 20 national | 480 counties + 160 states + 40 national |
| 30 | Nigeria | [49], [73] | 31 states + national level | 12 states + 20 national | 744 states + 40 national |
| 31 | Norway | [49], [74] | 18 states + national level | 16 states + 20 national | 576 states + 40 national |
| 32 | Pakistan | [49], [69] | 5 states + national level | 19 states + 20 national | 190 states + 40 national |
| 33 | Paraguay | [49], [75] | 19 states + national level | 19 states + 20 national | 722 states + 40 national |
| 34 | Peru | [49], [76] | 26 states + national level | 20 states + 20 national | 1040 states + 40 national |
| 35 | Philippines | [49], [69] | 11 states + national level | 19 states + 20 national | 418 states + 40 national |
| 36 | Poland | [49], [54] | 16 states + national level | 13 states + 20 national | 416 states + 40 national |
| 37 | Portugal | [49], [54] | 7 states + national level | 20 states + 20 national | 280 states + 40 national |
| 38 | Romania | [49], [54] | 8 states + national level | 14 states + 20 national | 224 states + 40 national |
| 39 | Saudi Arabia | [49], [77] | 14 states + national level | 20 states + 20 national | 560 states + 40 national |
| 40 | Slovakia | [49], [54] | 4 states + national level | 14 states + 20 national | 112 states + 40 national |
| 41 | South Africa | [49], [78] | 11 states + national level | 4 states + 20 national | 88 states + 40 national |
| 42 | Spain | [49], [54] | 16 counties + 6 states + national level | 20 counties + 20 states + 20 national | 640 counties + 240 states + 40 national |
| 43 | Sri Lanka | [49], [79] | 24 counties + 9 states + national level | 20 counties + 20 states + 20 national | 960 counties + 360 states + 40 national |
| 44 | Sweden | [49], [54] | 8 states + national level | 20 states + 20 - national | 320 states + 40 national |
| 45 | Thailand | [49], [80] | 72 counties + 5 states + national | 14 counties + 14 states + 20 - national | 2016 counties + 140 states + 40 national |
| 46 | Turkey | [49], [81] | 73 states + national level | 18 states + 20 national | 2628 states + 40 national |
| 47 | United Kingdom | [49], [54] | 12 counties + 4 states + national level | 20 counties + 20 states + 20 national | 480 counties + 160 states + 40 national |
| 48 | United States | [49], [82] | 3078 counties + 50 states + national level | 20 counties + 20 states + 20 national | 123120 counties + 2000 states + 40 national |
| 49 | Uruguay | [49], [83] | 19 states + national level | 2 states + 20 national | 76 states + 40 national |
| 50 | Venezuela | [49], [84] | 24 states + national level | 1 states + 20 national | 48 states + 40 national |
| 51 | Vietnam | [49], [85] | 61 counties + 8 states + national level | 14 counties + 14 states + 20 national | 1708 counties + 224 states + 40 national |
| Countries with incomplete national data | | | | | |
|  | Former Soviet Republics | [49], [86] | 1 national level | 19 – national (1989-1990; 1992-2008) | 38 national |
|  | Eritrea, Ethiopia | [49] | 1 national level | 20 national | 40 national |
| Countries with complete National data for all years (1989-2008) | | | | | |
| Afghanistan, Albania, Algeria, American Samoa, Andorra, Angola, Anguilla, Antigua and Barbuda, Aruba, Bahamas, Bahrain, Bangladesh, Barbados, Belize, Benin, Bermuda, Bhutan, Botswana, British Virgin Islands, Brunei Darussalam, Burkina Faso, Burundi, Ivory Coast, Cambodia, Cameroon, Cape Verde, Cayman Islands, Central African Republic, Chad, Republic of Congo, Democratic Republic of Congo, Denmark, Cook Islands, Costa Rica, Cuba, Cyprus, Democratic People’s Republic of Korea (North Korea), Djibouti, Dominica, Dominican Republic, Egypt, El Salvador, Equatorial Guinea, Faeroe Islands, Falkland Islands, Fiji Islands, French Guiana, French Polynesia, Gabon, Gambia, Ghana, Gibraltar, Greenland, Grenada, Guadeloupe, Guam, Guatemala, Guinea, Guinea-Bissau, Guyana, Haiti, Honduras, Iceland, Iraq, Isle of Man, Israel including Palestine, Jamaica, Jordan, Kenya, Kiribati, Kuwait, Lao People’s Democratic Republic, Lebanon, Lesotho, Liberia, Libyan Arab Jamahiriya, Liechtenstein, Luxembourg, Madagascar, Malawi, Malaysia, Maldives, Mali, Malta, Marshall Islands, Martinique, Mauritania, Mauritius, Mayotte, Micronesia (Federated States of), Monaco, Montserrat, Morocco, Mozambique, Myanmar, Namibia, Nauru, Netherlands Antilles, New Caledonia, New Zealand, Nicaragua, Niger, Niue, Norfolk Island, Northern Mariana Is, Oman, Palau, Panama, Papua New Guinea, Paracel Islands, Pitcairn Islands, Puerto Rico, Qatar, Reunion, Rwanda, Saint Helena, Saint Kitts and Nevis, Saint Lucia, Saint Pierre & Miquelon, Saint Vincent/Grenadines, Samoa, San Marino, Sao Tome and Principe, Senegal, Seychelles, Sierra Leone, Singapore, Solomon Islands, Somalia, Spratly Islands, Sudan, Suriname, Swaziland, Switzerland, Syrian Arab Republic, Tanzania, Togo, Tokelau, Tonga, Trinidad and Tobago, Tunisia, Turks and Caicos Is, Tuvalu, Uganda, United Arab Emirates, US Virgin Islands, Vanuatu, Wake Island, Wallis and Futuna Is, Western Sahara, Yemen, Zambia, Zimbabwe. | | | | | |
| Countries not analysed | | | | | |
| Bosnia and Herzegovina, Croatia, Serbia and Montenegro, Slovenia, Taiwan | | | | | |
