# Supplementary material for: Yield Trends Are Insufficient to Double Global Crop Production by 2050
Source: PLoS One. 2013 Jun 19;8(6):e66428. doi: 10.1371/journal.pone.0066428 (PMC3686737; doi:10.1371/journal.pone.0066428)
Supplement: Table S2 — Current yields, projections and production for the Brazilian Legal Amazon. (DOCX) [file pone.0066428.s014.docx]

**Ray et al. Supporting Information Table S2.** Current yields, projections and production for the Brazilian Legal Amazon

|  | MAIZE | RICE | WHEAT | SOYBEAN |
| --- | --- | --- | --- | --- |
| Mean yield change per year (%/year) | 2.6 | 2.8 | 1.6 | 1.5 |
| Mean yield change per year (kg/ha/year/year) | 83.0 | 92.4 | 19.7 | 44.3 |
| Projected average yield in 2025 (tons/ha/year) | 4.6 | 4.9 | 1.6 | 3.7 |
| Projected production in 2025 (million tons/year) | 11.5 | 4.7 | 0.3 | 20.7 |
| Yield in the year 2008 (tons/ha/year) | 3.6 | 3.4 | 1.4 | 2.9 |
| Production in the year 2008 (million tons/ year) | 9.2 | 3.3 | 0.2 | 16.3 |
