# Supplementary material for: Yield Trends Are Insufficient to Double Global Crop Production by 2050
Source: PLoS One. 2013 Jun 19;8(6):e66428. doi: 10.1371/journal.pone.0066428 (PMC3686737; doi:10.1371/journal.pone.0066428)
Supplement: Table S3 — Comparison with the future U. S. crop yields reported by the USDA-ERS [91] (maize, wheat, and soybean were reported in bushels per acre, rice in pounds per acres from USDA-ERS and converted to ton/ha), and global wheat yields reported by the FAO-OECD [90]. (DOCX) [file pone.0066428.s015.docx]

**Ray et al. Supporting Information Table S3.** Comparison with the future U. S. crop yields reported by the USDA-ERS [91] (maize, wheat, and soybean were reported in bushels per acre, rice in pounds per acres from USDA-ERS and converted to ton/ha), and global wheat yields reported by the FAO-OECD [90].

| Year | Maize | | | Rice | | | Wheat | | | | | | Soybean | | |
| --- | --- | --- | --- | --- | --- | --- | --- | --- | --- | --- | --- | --- | --- | --- | --- |
|  | USDA (t/ha) | This study (t/ha) | % diff | USDA (t/ha) | This study (t/ha) | % diff | USA only | | | Global | | | USDA (t/ha) | This study (t/ha) | % diff |
|  |  |  |  |  |  |  | USDA (t/ha) | This study (t/ha) | % diff | FAO-OECD | This study | % diff |  |  |  |
| 2009 | 9.87 | 9.81 | 0.65 | 8.16 | 7.89 | -3.31 | 2.88 | 2.96 | 2.80 |  |  |  | 2.87 | 2.81 | -1.83 |
| 2010 | 10.0 | 9.96 | 0.42 | 8.24 | 7.99 | -3.16 | 2.90 | 2.99 | 3.03 |  |  |  | 2.89 | 2.84 | -1.75 |
| 2011 | 10.13 | 10.10 | 0.19 | 8.32 | 8.06 | -3.02 | 2.92 | 3.01 | 3.25 |  |  |  | 2.93 | 2.87 | -1.89 |
| 2012 | 10.25 | 10.25 | -0.03 | 8.39 | 8.15 | -2.81 | 2.94 | 3.04 | 3.47 | 3.1 | 3.0 | -3.23 | 2.95 | 2.90 | -1.80 |
| 2013 | 10.38 | 10.40 | -0.25 | 8.46 | 8.24 | -2.59 | 2.96 | 3.07 | 3.70 | 3.1 | 3.1 | 0.00 | 2.99 | 2.93 | -1.94 |
| 2014 | 10.50 | 10.55 | -0.46 | 8.53 | 8.32 | -2.43 | 2.98 | 3.10 | 3.91 | 3.2 | 3.1 | -3.13 | 3.01 | 2.96 | -1.86 |
| 2015 | 10.63 | 10.70 | -0.66 | 8.59 | 8.41 | -2.17 | 3.00 | 3.12 | 4.12 | 3.2 | 3.1 | -3.13 | 3.05 | 2.99 | -1.99 |
| 2016 | 10.75 | 10.85 | -0.86 | 8.66 | 8.49 | -1.93 | 3.02 | 3.15 | 4.33 | 3.2 | 3.1 | -3.13 | 3.07 | 3.01 | -1.91 |
| 2017 | 10.88 | 10.99 | -1.06 | 8.72 | 8.58 | -1.70 | 3.04 | 3.18 | 4.54 | 3.2 | 3.2 | 0.00 | 3.11 | 3.04 | -2.04 |
| 2018 |  |  |  |  |  |  |  |  |  | 3.3 | 3.2 | -3.03 |  |  |  |
| 2019 |  |  |  |  |  |  |  |  |  | 3.3 | 3.2 | -3.03 |  |  |  |
| 2020 |  |  |  |  |  |  |  |  |  | 3.3 | 3.3 | 0.00 |  |  |  |
| 2021 |  |  |  |  |  |  |  |  |  | 3.3 | 3.3 | 0.00 |  |  |  |
| 2022 |  |  |  |  |  |  |  |  |  | 3.4 | 3.3 | -2.94 |  |  |  |
