# Supplementary material for: Yield Trends Are Insufficient to Double Global Crop Production by 2050
Source: PLoS One. 2013 Jun 19;8(6):e66428. doi: 10.1371/journal.pone.0066428 (PMC3686737; doi:10.1371/journal.pone.0066428)
Supplement: Text S1 — Additional Data, Model Fitting, Rates, and Inter-comparison information. (DOC) [file pone.0066428.s016.doc]

**Ray et al. Supporting Information Text S1**

*Data*

We use ~2.5 million reported maize, rice, wheat, and soybean area harvested and yield observations between 1961-2008 from the database reported in [23] focusing on the period 1989 to 2008 using ~1.8 million crop reports. Data are from 13,500 fixed political units. We track changes annually in these two crop metrics sub-nationally for 51 major agricultural countries, currently responsible for 70% to 90% of the global harvested areas and 79% to 99% of total global production in these four crops [49]. Seventeen of these countries are tracked at the sub-state / sub-province, i.e. county / district level. Statistics are tracked nationally for the remaining countries. Supporting Table S1 provides further details on the number of political units tracked per country, length of time for which census statistics are available per country, and total number of statistics collected per crop per country for the period 1989 to 2008.

The raw statistics collected were first inspected and quality controlled (e.g. any obvious errors removed, or corrected). For example we found reported statistics such as 57.9 tons/ha/year of rice yield in a province of Indonesia for year 2007, when all pre- and post- reported yields were less than 10 tons/ha/year. We corrected this data by using the yield information that was present together with the production numbers to correct for the harvested hectares; harvested hectares and production were the raw statistics collected and yield was the derived quantity. After quality controlling, all data were converted to the same units of, hectares for harvested areas, and tons/ha/year for yields.

The frequency of reported sub-national data varies by country (Supporting Table S1) and is generally more available from recent years. When data is unavailable for specific years we interpolate using agricultural statistics available from the larger political unit, within which the higher resolution political unit is nested, in three distinct steps. First, we identified the nearest five years of available data years. Next, the five-year average proportions of harvested areas and production numbers of the units within the higher political unit are calculated. Using these proportions the higher political units harvested areas and production numbers are disaggregated for the missing year in the third step. Finally, yields are computed.

The number of interpolated data are not included in the ~1.8 million number reported above. In cases where there is a discrepancy between the total of subnational reported statistics summarized to the national scale, and the Food and Agriculture Organization (FAO) reported numbers [49], the subnational numbers are proportionately scaled to match the FAO statistics.

*Model fitting*

In order to determine the current rates of yield change, two methodological decisions needed our careful consideration: (1) what statistical model fits should be used to determine the rates of yield change; and (2) what length of time analyzed would capture the current rates of crop yield change best.

Consistent with previous studies, we utilized simple linear regression (Eqn. 1) to track yield trends. Here we linearly regressed the observed maize, rice, wheat, and soybean crop yields against time to determine the non-compounding linear rates of yield change in each of the ~13,500 political units.

(1)

where

*Y* = yield in tons/ha/year,

*t* = year,

*a* is the coefficient of regression and is the rate term (tons/ha/year/year),

*k* is the intercept term of the linear regression, and

*e* is the error term.

We use this approach to compute the linear, non-compounding rates of yield change over the 1989 to 2008 observed time period. We then project the yields annually to the year 2050 in order to determine the production consequences were these average rates of change to continue. Rates for each crop and country are reported in the Supporting Data file. The coefficient of variation (r^2^) associated with the 20 year data analysis is provided in Supporting Figure S1. As some political units violate assumptions of simple linear regression for determining normal confidence intervals (as we discuss below), we use 99 bootstrapped samples for each political unit over this two-decade observation period to determine a 90 percent confidence interval for the linear rates of yield change. These high and low estimates on the linear rates of change are used to set the high and low projections in the main text (Figure 1 in the Main Text).

With 13,500 political units, not all of the yield time series met assumptions about autocorrelation or normality. Yearly crop yield observations can generally be regarded as independent observations, as each growing season represents a new cycle of planting, weather, and crop management. However, it is possible autocorrelation between years may arise due to longer-term weather cycles or periods of technology diffusion [87]. For transparency of where these issues arise, we conduct the Lilliefors test for normality (Supporting Figure S2) and the Durbin-Watson test for autocorrelation (Supporting Figure S3) at each political unit and crop for which we have data.

Consider the following two examples of model fits to understand these statistical results further: national soybean yields in the United States (Supporting Figure S4) and national maize yields in Angola (Supporting Figure S5). Our dataset does contain subnational United States soybean yields, but we show the national data here as it is freely available from FAO. United States soybean yields meet normality, constant variance, and autocorrelation assumptions when fit with a linear model (Supporting Figure S4). Angola’s maize yields meet normality assumptions but violate assumptions for autocorrelation and non-constant variance when fitted with a linear model (Supporting Figure S5).

For comparison, we also conducted a separate analysis at each political unit, choosing the best statistical model by parsimoniously fitting polynomials of increasing order using the Akaike Information Criterion (AIC) as the determining factor [88]. Although this approach leads to better regression fits in some political units, unfortunately the higher order polynomials thus selected have strong acceleration terms (higher orders of time). These strong acceleration terms induce unrealistic rapid yield changes over a short period of only a few years (Supporting Figure S6). In the case of Angola maize yields, AIC chooses a quadratic model (Supporting Figure S7), which alleviates concern over non-constant variance and autocorrelation seen in the linear model; however the quadratic model projects negative maize yield in Angola by 2050 (Supporting Figure S8).

As illustrated by these examples and Supporting Figures S2-S8, simple linear regression may not always be the most appropriate statistical model over the time period of observations for all of the political units. However, higher-order polynomials are dangerous for extrapolating into the future. As our goal here is to identify the average linear rates of change over the recent past, and discuss the consequences were these average rates of change were to continue into the future, we adhere to simple linear regression for this analysis. This decision aids ease of interpretation and is consistent with many other time-yield analyses (as noted in the main text).

We found, as expected, that the determination of current rates was sensitive to the number of years analyzed. When fewer years are analyzed the calculated rates are strongly influenced by shorter-term shocks to yields. On the other hand, calculating rates across several decades of observed yields does not reflect recent trends. As there is no prescribed scientific procedure to deduce *a-priori* the correct number of years to conduct such an analysis, we tested several time spans and found that two decades (1989-2008) of yield data generally reduced both the effects of short-term shocks and captured the current rates. This window of time has also been used in other studies [28]. Performing the analysis using fifteen and twenty-five years of data resulted in only slightly different results (Supporting figure S9); as the number of years analyzed increases or decreases from these number of years, the results increasingly diverge. As a result, we restricted our analysis to the most recent 20 years (1989 to 2008) instead of the entire data from 1961 to 2008.

The crop yields in the last year of our observation period, (2008), influences the percentage rate of growth, but not the real rates of yield increases in kg/ha/year/year (Supporting Figure S10). We note the spatial variations in the yields (tons/ha/year) of these four crops in the year 2008 in Supporting Figure S11.

*Summarizing rates and yields to higher spatial units*

When we summarize results to a higher political unit such as the country level or global level from subnational political units we first summarize the observed total harvested area and production for all the political units in a country (or all the political units globally when providing global numbers). We next determine the area weighted observed and bootstrapped yields at the higher political unit. The linear fit to these yields at the country or global level using equation 1 provides the linear rates of observed yield change. We project these rates forward at the country or global level. We similarly determine the rates associated with the confidence interval. Thus, while we provide the subnational changes in crop yields, our summary at the national scale and global scale would be nearly identical to those studied using only the national or global scale yield data. The Supporting Data file contains rates for the major countries that harvested maize, rice, wheat, and soybean.

Subnational level analysis has the advantage of summarizing rates of yield change at other unique levels such as hydrological watersheds, ecoregions, climatic zones, mesoregions within a country. Supporting Figure S12 and Supporting Table S2 provide an example of using these data to assess yield trends within the Brazilian Legal Amazon, the watershed comprising the Amazon and Tocantins rivers in Brazil. Our analysis shows that soybean yields are changing at 1.5%/year (44.3 kg/ha/year/year) in the Brazilian Legal Amazon area and at this rate would reach 3.7 tons/ha/year in 2025 from 2.9 tons/ha/year in 2008. This increased yield would lead to soybean production of 20.7 million tons/year in 2025 in the Brazilian Legal Amazon from the 16.3 million tons/year in 2008.

# *Inter-comparison with other results*

We compared our projected global yield with the FAO [89] reported global yield in the year 2009-2010; they were very similar, as can be expected from a projection that is only 1-2 years out into the future. Longer-term global projections from the FAO-OECD [90] summarize yields over all cereal crops and thus are not directly comparable to our individual crop results. For wheat however projections are reported from the FAO-OECD [64] and we found them similar to our projections (Supporting Table S3), though it should be noted that the FAO-OECD yield projections include the effects of projected future changes in harvested areas and also unknown expert-driven adjustments and methodologies [90]; the difference with our projections was minor, a maximum difference of 3% for a few years (Supporting Table S3).

We were able to acquire longer time scale crop forecasts such as presented in this article, only from the United States, which freely disseminates its crop yield projection. The USDA-ERS [91] 10-year projections for several agricultural sector commodities (including those studied here) are based on two years of history and “reflect a composite of model results and judgment-based analysis” [91]. The difference between from our projections are no more than -3.3% to +4.5% for the four crops and all future years. Supporting Table S3 lists the USDA-ERS ten-year crop yield projections and the difference with our projection for the U. S.

References

49. FAO, Food and Agricultural Organization, http://faostat.fao.org, accessed regularly between 2010 and 2012.

50. Sistema Integrado de Informacion Agropecuaria, Argentina, http://www.siia.gov.ar/, accessed March 2011.

51. Australian Natural Resources Atlas, http://www.anra.gov.au/, accessed June 2011.

52. Australian Bureau of Statistics, http://www.abs.gov.au, accessed January 2012.

53. Australian Oilseeds Federation Annual Reports.

54. Eurostat, http://epp.eurostat.ec.europa.eu/portal/page/portal/statistics/search_database, accessed November 2010.

55. Instituto Nacional de Estadistica, Bolivia, http://www.ine.gob.bo/, accessed February 2011.

56. Instituto Brasileiro de Geografia e Estatistica, Brazil, http://seriesestatisticas.ibge.gov.br/Default.aspx, accessed between 2011 and 2012.

57. Anuario Estatistico do Brasil, Volumes 22-52.

58. Statistics Canda, http://www.statcan.gc.ca/start-debut-eng.html, accessed March 2010.

59. Oficina de Estudios Y Politicas Agrarias, Chile, http://www.odepa.gob.cl, accessed March 2011.

60. USDA Economic Research Service, http://www.ers.usda.gov/data/china/, accessed April 2010.

61. Ministerio de Agricultura y Desarrollo Rural - Secretarías de Agricultura Departamentales, Colombia, http://www.dnp.gov.co/, accessed February 2011.

62. Czech Statistical Office, http://www.czso.cz/eng/redakce.nsf/i/home, accessed January 2012.

63. Ministerio de Agricultura, Ganaderia Acuacultura y Pesca, http://www.magap.gob.ec/mag01/, accessed March 2011.

64. Chandok, H. L. and The Policy Group, India Database, The Economy, Annual Time Series Data, Vol II, 1990

65. Indiastat, http://www.indiastat.com/agriculture/2/stats.aspx, accessed February 2010.

66. Directorate of Rice, Wheat and Millet development, http://dacnet.nic.in/profile.asp, accessed in 2011.

67. Badan Pusat Statistik, http://dds.bps.go.id/eng/index.php, accessed February 2011.

68. Statistical Center of Iran, http://amar.sci.org.ir/index_e.aspx, accessed April 2011.

69. Regional Data Exchange System, http://faorap-apcas.org, accessed February 2011.

70. Korea Statistical Yearbooks (Hanʾguk tʻonggye yŏnʾgam).

71. Servicio de Informacion Agroalimentaria y Pesquera, http://www.siap.gob.mx/, accessed March-April, 2011.

72. National Statistical Office of Mongolia, http://www.nso.mn/v3/index2.php?page=free_access, accessed February 2011.

73. National Bureau of Statistics, Nigeria, http://www.nigerianstat.gov.ng/, accessed January 2011.

74. Statistics Norway, http://www.ssb.no/english/, accessed August 2011.

75. Ministerio de Agricultura y Ganaderia, http://www.mag.gov.py/, accessed February 2011.

76. Ministerio de Agricultura, http://frenteweb.minag.gob.pe/, accessed February 2011.

77. Central Department of Statistics and Information, Kingdom of Saudi Arabia, http://www.cdsi.gov.sa/english/index.php, accessed May 2011.

78. Agriculture census, South Africa, http://www.harvestchoice.org and http://www.statssa.gov.za/agriculture/default.asp, accessed March 2011.

79. Department of Census and Statistics, http://www.statistics.gov.lk/agriculture/index.htm, accessed February 2011.

80. National statistical office, Thailand, http://web.nso.go.th/, accessed February 2011.

81. Turkiye Istatistik Kurumu, http://www.tuik.gov.tr/Start.do, accessed February 2011.

82. National Agricultural Statistics Service, United States Department of Agriculture, http://quickstats.nass.usda.gov/, accessed September 2011.

83. Censo general Agropecuario 1980, 2000. Uruguay.

84. Agriculture Censos 1961, 1971, Venezuela.

85. General Statistics Office of Vietnam, http://www.gso.gov.vn/default_en.aspx, accessed February 2011.

86. The World Bank, Statistical handbook States of the former USSR, 1992.

87. Luttrell CB, and Gilbert RA (1976) Crop Yields: Random, Cyclical, or Bunchy? Agricultural and Applied Economics Association 58(3): 521-531.

88. Akaike, H (1974) A new look at the statistical model identification. IEEE Transactions on Automatic Control 19: 716–723.

89. Food and Agriculture Organization of the United Nations (FAOSTAT). http://faostat.fao.org/site/567/default.aspx#ancor (accessed, March 2011).

90. OECD-FAO Agricultural Outlook 2012-2021. http://www.oecd.org/site/oecd-faoagriculturaloutlook/ (accessed October 2012).

91. United States Department of Agriculture, Economic Research Service, http://www.ers.usda.gov/ (accessed September 2012).
